# Antimicrobial activity and antifungal mechanistic study of 3‑substituted oxindoles against *Aspergillus niger*

**DOI:** 10.1038/s41598-025-27782-4

**Published:** 2025-11-29

**Authors:** Hend A. A. Ezelarab, Maisra M. El-Bouseary, Ramadan Yahia, Rehab Mahmoud Abd El-Baky, Mohamed A. Mawhoup, Eman Farouk Ahmed, Ghada M. Sadiq, Taha F. S. Ali, Samar H. Abbas, Heba A. Hassan, Eman A. M. Beshr

**Affiliations:** 1 Department of Medicinal Chemistry, Faculty of Pharmacy, Minia University, Minia, 61519 Egypt; 2 Medicinal Chemistry Department, Faculty of Pharmacy, Minia National University, New Minia, Egypt; 3 Department of Microbiology and Immunology, Faculty of Pharmacy, Tanta University, Tanta, Gharbia, Egypt; 4 Microbiology and Immunology Department, Faculty of Pharmacy, Badr University in Assiut, Assiut, 77771 Egypt; 5 Microbiology and Immunology Department, Faculty of Pharmacy, Minia University, Minia, 61519 Egypt; 6 Microbiology and Immunology Department, Faculty of Pharmacy, Deraya University, Minia, Egypt; 7 Department of Microbiology, Faculty of Pharmacy, Sohag University, Sohag, Egypt; 8 Department of Pharmaceutical Chemistry, Faculty of Pharmacy, Deraya University, Minia, Egypt; 9 Pharmaceutical Chemistry Department, Faculty of Pharmacy, Badr University in Assiut (BUA), Assiut, Egypt

**Keywords:** 3-Substituted oxindole, Antifungal activity, *Aspergillus niger*, Chitin deacetylase AngCDA, 1,3-Beta-glucan synthase, Biochemistry, Biotechnology, Drug discovery, Microbiology

## Abstract

A series of 3-substituted oxindole derivatives **3a-j** was synthesized. The target compounds **3a-j** were investigated for their potential antibacterial and antifungal activities. *S. aureus, MRSA, E. faecalis, P. aeruginosa, K. pneumonia,* and *E. coli* were used for testing the antibacterial activity, while *Candida albicans and Aspergillus spp,* were used for antifungal activity of the tested compounds. Compounds **3i** and **3j** exhibited the strongest activity, particularly against Gram-positive bacteria. Antifungal activity was tested against *Aspergillus spp*. and *Candida albicans* using the agar diffusion method, with a final concentration of 10,000 µg/mL per well. Eight compounds inhibited *Aspergillus spp.,* while only compound **3g** was active against *C. albicans*. Compound **3f** displayed the highest antifungal activity (inhibition zone = 20 mm) compared with itraconazole with a final concentration of 100 µg/mL per well (30 mm). Minimum inhibitory concentration (MIC) testing confirmed compound **3f** as the most potent antifungal, with an MIC of 7.5 µg/mL against *Aspergillus niger*, lower than that of clotrimazole (12.5 µg/mL). Further investigations showed that compound **3f** exerted its effect by disrupting fungal cell wall integrity without binding ergosterol. Docking studies of compound **3f** in the active sites of chitin deacetylase AngCDA (PDB ID: 7BLY) and the 1,3-β-glucan synthase (8JZN) enzymes proved its dual-target mechanism ability to inhibit the cell wall biosynthesis. These findings highlight compound **3f** as a promising lead scaffold for the development of new antifungal agents targeting fungal cell wall biosynthesis.

**Supplementary Information:**

The online version contains supplementary material available at 10.1038/s41598-025-27782-4.

## Introduction

The incidence and prevalence of invasive fungal infections have risen in recent years, particularly among the substantial population of immunocompromised patients and those hospitalized with severe underlying conditions. Fungal species account for 25% of microorganisms identified in blood cultures from hospitalized patients.

It is frequently observed in various cancer patients that concomitant fungal and bacterial infections occur^1^.

Consequently, the combination of antifungal drugs like Itraconazole with other anticancer and antibacterial agents, such as ciprofloxacin, yields more potent and efficient antifungal activity compared to the use of antifungal drugs alone^1^. Consequently, the development of innovative dual anticancer and antifungal drugs that exhibit enhanced efficacy, selectivity, and cost-effectiveness is essential for research scientists.

Species of *Aspergillus*, such as *A. fumigatus*, *A. flavus*, *A. niger*, *A. terreus*, and *A. versicolor*, are frequently associated with conditions including rhinosinusitis, cutaneous and subcutaneous aspergillosis, pulmonary infections, heart infections, and otomycosis, among others^1,2^. The genus generally shows limited ability to cause disease in humans and requires a significant amount of inoculum to infect individuals with compromised immune systems, assuming their physiological functions are otherwise normal. After the infection takes hold, various presentations are observed, with the lungs being the organ most significantly impacted^3^.

The infectious spectrum of aspergillosis encompasses invasive disease, leading to life-threatening infections in immunocompromised individuals; subacute or chronic infectious disease, impacting those with structural lung abnormalities, pre-existing sinus or lung conditions, or minor defects in innate immunity; and allergic or eosinophilic disease, which presents in various forms^1,4^.

The pathogenesis of aspergillosis is influenced by the immunological status of the host and the virulence of the pathogen. *Aspergillus species* possess potential virulence factors. Examples of adhesions include biofilm formation, haemolysin, pigment hydrolysis, and non-protein metabolites^5,6^. Virulence factors may play a role in the development of invasive aspergillosis^7,8^.

Fluconazole, Itraconazole, Voriconazole, and Amphotericin B are utilized in different formulations for the treatment of these infections. Fungal illness treatment is frequently ineffective due to azole resistance and the nephrotoxic and neurotoxic effects of Amphotericin B, raising concerns among health professionals^9^. Amidst global environmental changes and increasing vulnerable populations, pathogenic fungi that infect humans are developing resistance to all approved systemic antifungal agents. Four categories of systemically active antifungals—polyenes, azoles, echinocandins, and 5-flucytosine—have been predominant in treatment^10^. Fungi exhibit rapid responses to chemicals, which increases the likelihood of treatment failure. This failure results from underlying host immunological deficiencies, characteristics of antifungal medications (including pharmacokinetics, pharmacodynamics, and drug–drug interactions), and fungal attributes such as diverse cell morphologies, antifungal tolerance, and resistance. Alterations in drug–target interactions often lead to antifungal resistance. Resistance may arise from genetic alterations, such as mutations in lanosterol demethylase affecting azole efficacy and β-glucan synthase impacting echinocandin effectiveness^10^ Additionally, it can result from the overexpression of drug targets or changes in drug concentration, including enhanced efflux activity for intracellular agents and the inhibition of prodrug activation in flucytosine^11–13^.

**Fig. 1:**
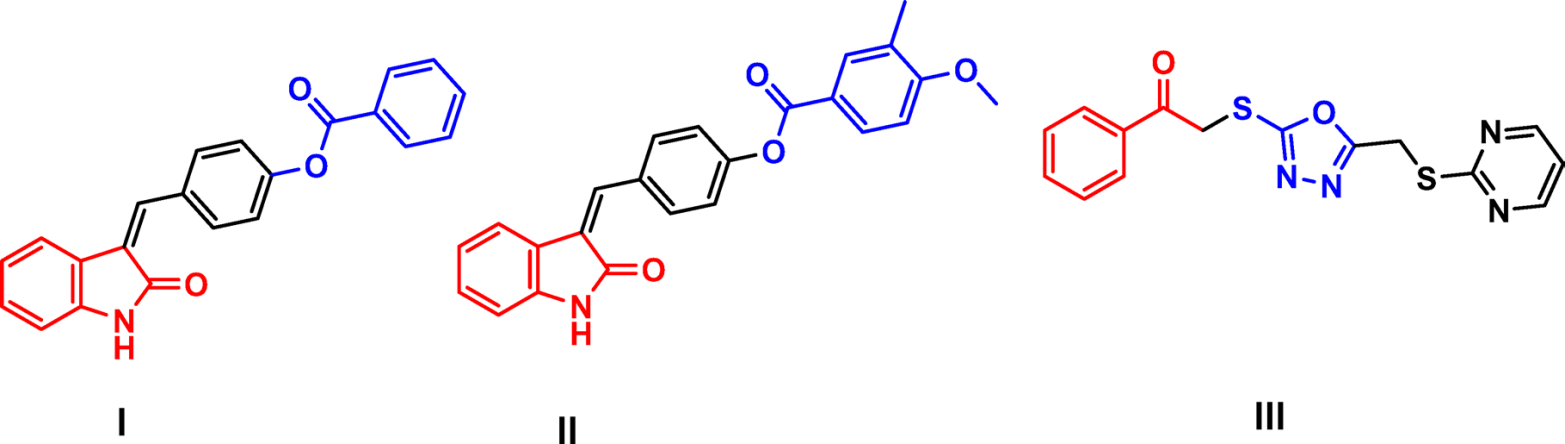
Structure of promising antimicrobial indole, and oxindole-based compounds (I-III).

At the same time, Indole-based and the 3-substituted oxindole scaffolds have been shown to have significant potential to be used in numerous biological applications such as antimicrobial, anticancer, antiviral, antileishmanial, antitubercular, antioxidative, and analgesic effects^14,15^. Moreover, substitution on carbon-4 and carbon-5 positions at 3-alkenyl indoles and/or oxindoles is preferred to be electronegative substituents such as halogens, mostly bromine and chlorine atoms, that are typically shown to good germicidal activity against Gram-positive (*Staphylococcus aureus*) bacteria, methicillin-resistant *Staphylococcus aureus* (*MRSA*), and against Gram-negative (*E. coli*) bacteria^15^. Based on prior literature data, analogues **I and II** (Fig. 1) with benzoyl and 3,4-dimethoxy benzoyl groups afforded modest antimicrobial activity against *M. tuberculosis* H37Rv with MIC values of 73 μM and 31 μM, respectively^16^. Additionally, compound **III**, a phenacyl-based scaffold, showed highly potent activity against all tested *Candida* classes except *C. tropicalis* and *C. krusei* with an MIC value of 0.007–0.06 mg/mL relative to the reference antifungal ketoconazole with an MIC value of 0.001–0.007 mg/mL (Fig. 1)^17^.

To address the aforementioned issues, it is essential to explore novel therapeutic strategies for resistant fungal strains. This may involve the use of combinations of two antifungal agents, evaluating the synergistic effects of non-antimicrobials in conjunction with antifungals, or investigating new alternatives.

**Fig. 2:**
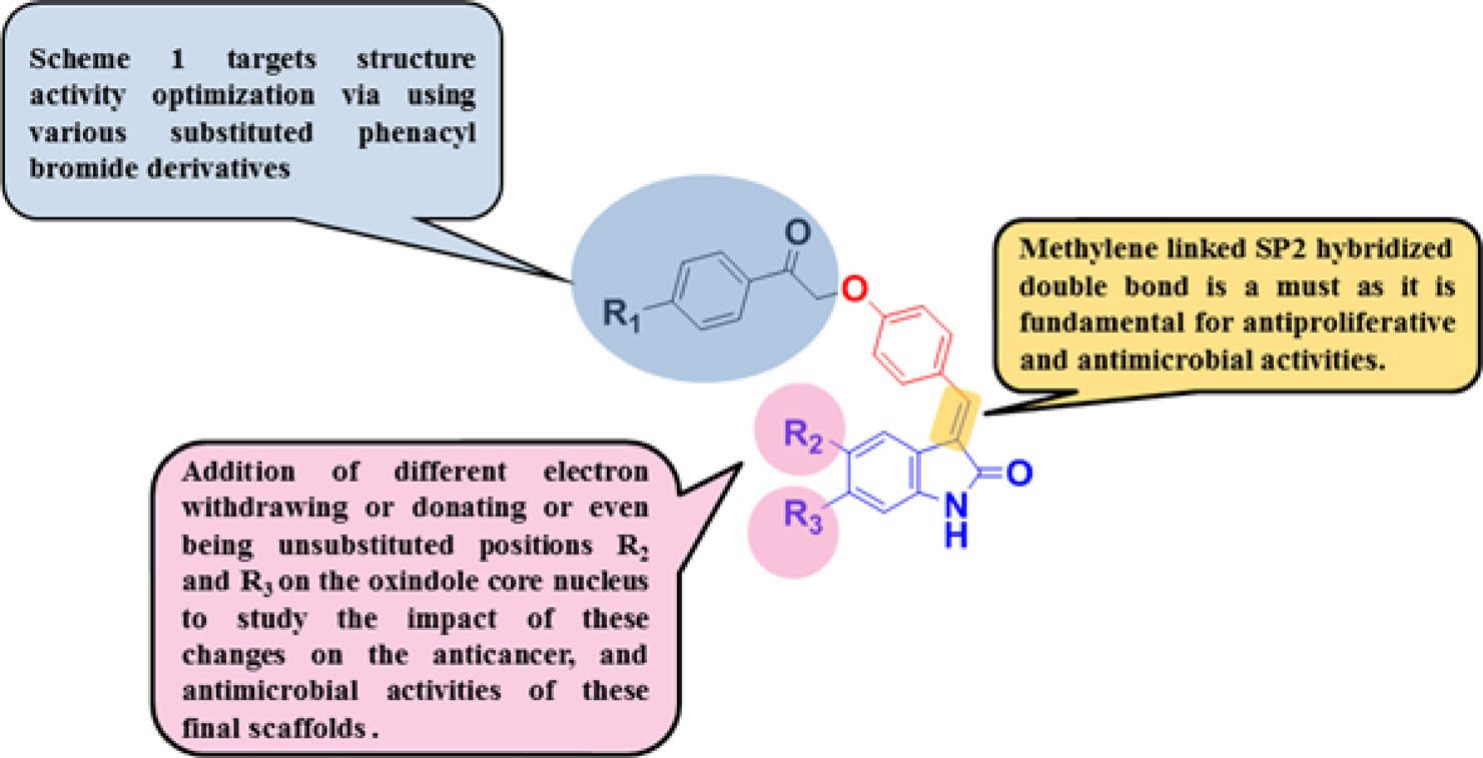
Rationalized design of scaffolds 3a-3j as anticancer and antimicrobial agents.

This study aims to prepare and evaluate the antimicrobial and antifungal activities of the antiproliferative compounds **3a-3j** (Fig. 2) by measuring their inhibition zone diameters against Gram-positive bacteria (*Staphylococcus aureus* (ATCC29213), *Enterococcus faecalis*, and *Methicillin-Resistant Staphylococcus aureus* (*MRSA*)), Gram-negative bacteria (*E. coli* (ATCC8729), *Klebsiella pneumonia*, and *Pseudomonas aeruginosa*), *and Candida albicans,* and *Aspergillus spp*. The minimum inhibitory concentration (MIC) and the potential mechanism underlying the antifungal activity of the most effective anti-Aspergillus agent were evaluated against *Aspergillus niger*. The most effective anti-aspergillus derivative was docked into the active sites of chitin deacetylase AngCDA (PDB ID: 7BLY) and 1,3-β-glucan synthase (8JZN).

## Results and discussion

### Chemistry

**Fig. 3:**
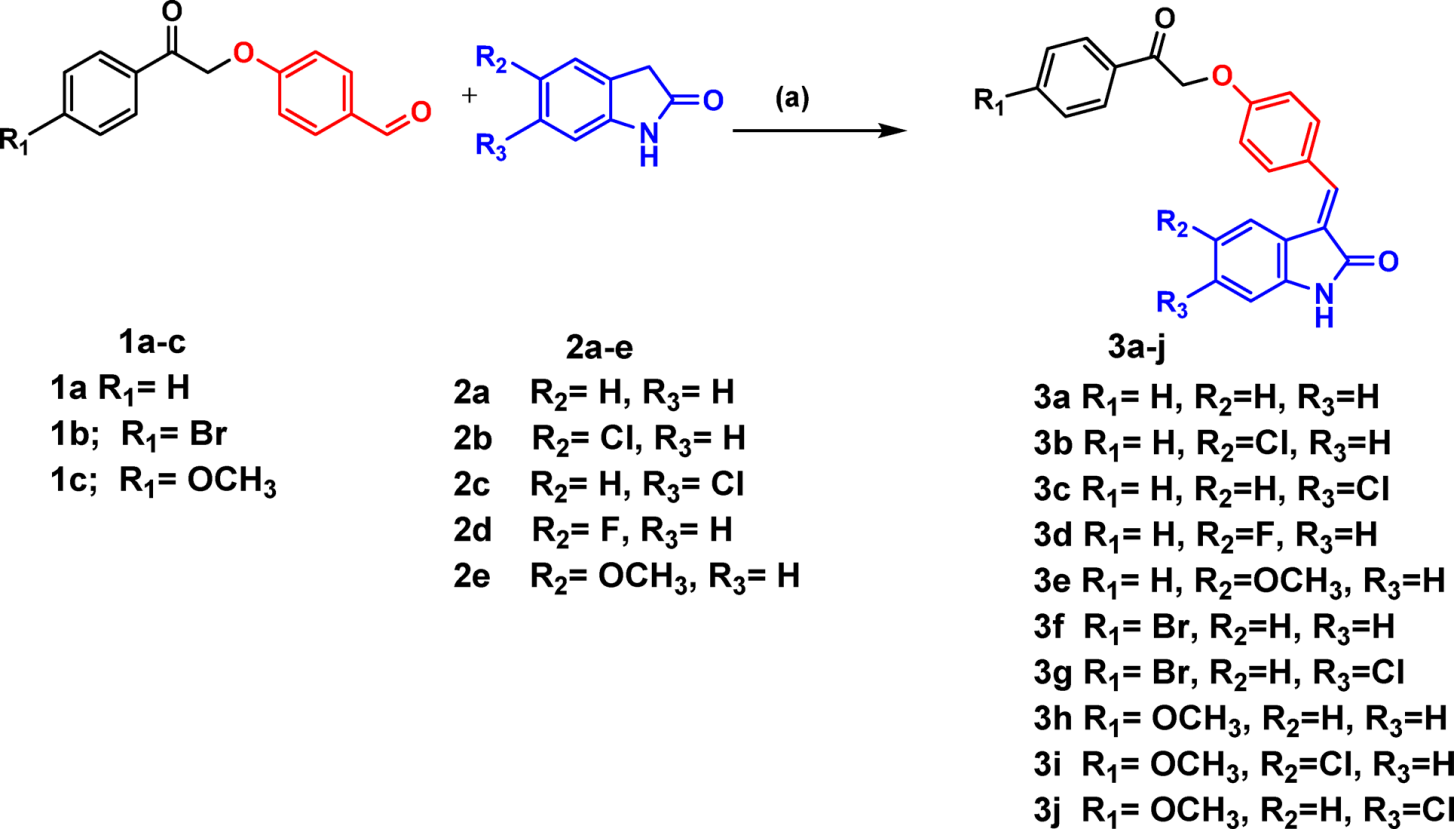
Synthesis of target oxindole derivatives 3a-j. Reagents: (**a**) Piperidine, EtOH, reflux 24 h.

The target oxindole derivatives **3a-j** were synthesized as previously reported^18^ by base-catalyzed Knoevenagel condensation reaction of the alkylated *p*-hydroxy benzaldehyde derivatives **1a-c** with the oxindole derivatives **2a-e** in the presence of piperidine as catalyst to afford compounds **3a-j** in a good yield (supporting materials), (Fig. 3).

### Screening of antimicrobial activity of compounds 3a-j

#### Screening of antibacterial activity

The antibacterial activity of scaffolds **3a-j** was tested in vitro against the Gram-positive bacteria (*Staphylococcus aureus* (ATCC29213), *Enterococcus faecalis*, and Methicillin-Resistant *Staphylococcus aureus* (*MRSA*)) and Gram-negative bacteria (*E. coli* (ATCC8729), *Klebsiella pneumonia*, and *Pseudomonas aeruginosa*), *Candida albicans,* and *Aspergillus spp*. (*MRSA*), Gram-negative bacteria (E. coli (ATCC8729), *Klebsiella pneumonia*, and *Pseudomonas aeruginosa*), compared with ciprofloxacin and streptomycin as antibacterial references using the cup plate agar diffusion method^19^.

Table 1 showed that compounds **3i** and **3j** showed the highest antibacterial activity among the other tested compounds. Compound **3i** inhibited Gram-positive bacteria with inhibition zone diameters (IZDs) of 13 mm (*S. aureus*), 13 mm (*MRSA*), and 14 mm (*E. faecalis*). Compound **3j** exhibited broader activity, inhibiting both Gram-positive (*S. aureus*, IZD = 15 mm; *MRSA,* IZD = 16 mm) and Gram-negative *(P. aeruginosa*, IZD = 11 mm) bacteria.

**Table 1:** Antimicrobial activity of compounds 3a-3j using the final concentration of each compound (1 × 10^4^ μg/mL) in one hole compared to the standards (Ciprofloxacin and Itraconzole) with a final concentration (1 × 10^2^ μg/mL) in one hole.

Inhibition zone diameter (mm)									
Tested compound	Tested bacterial isolates						Tested fungal isolates		*cLogP*
	Gram-positive bacteria			Gram-negative bacteria					
	*S. aureus*	*MRSA*	*E. faecalis*	*E. coli*	*P.* aeruginosa	*K. pneumoniae*	*C. albicans*	*Aspergillus* spp.	
3a	–	–	–	–	–	–	–	**16**	3.76
3b	–	–	–	–	–	–	–	**15**	4.41
3c	–	–	–	–	–	–	–	**17**	4.41
3d	–	–	–	–	–	–	–	**17**	4.32
3e	–	–	–	–	–	–	–	**15**	3.77
3f	–	–	–	–	12	–	–	**20**	4.52
3g	–	–	12	–	–	–	**12**	–	5.18
3h	13	–	–	–	12	–	–	–	3.77
3i	13	13	14	–	–	–	–	12	4.42
3j	**15**	**16**	–	–	11	–	–	14	4.42
Ciprofloxacin	**28**	**24**	**32**	**37**	**44**	**16**	–	–	1.18
Itraconazole	–	–	–	–	–	–	**28**	**30**	4.71

Significant values are in bold.

Regarding the partition coefficients of the tested compounds, we reported that the cLogP values of compounds **3i** and **3j** were 4.42 (as shown in Table 1). So, they showed greater lipophilicity than ciprofloxacin (1.18). This property may explain their higher activity against Gram-positive bacteria, since lipophilic compounds diffuse more effectively across the thick peptidoglycan layers lacking the outer membrane barrier characteristic of Gram-negative organisms^20,21^. The diffusion of lipophilic molecules through the lipid bilayer is slowed by the outer membrane^22^. This is consistent with previous studies, which reported that the most active compounds against Gram-negative bacteria are generally less lipophilic than those active against Gram-positive ones^23–26^. For this reason, the lipophilicity of a compound is a critical feature closely related to its antibacterial activity.

Moreover, it was found that compounds **3h-3j** displayed promising antibacterial activity against Gram-positive *S. aureus* bacteria with IZD values of 13 mm, 13 mm, and 15 mm, respectively, compared to reference positive control ciprofloxacin with an inhibition zone diameter of 28 mm (as shown in Table 1). Consequently, it can be concluded that the presence of an electron-donating function group at the *para* position of the phenacyl moiety, besides position 5 or 6 of oxindole, being unsubstituted or even substituted with electron-withdrawing Cl atoms, is fundamental to afford good antibacterial activity against Gram-positive *S. aureus* bacteria. Moreover, it was found that compounds **3i** and **3j** exhibited good antibacterial activity against Gram-positive *MRSA* with inhibition zones’ diameters of 13 mm and 16 mm, respectively, compared to ciprofloxacin with an inhibition zone diameter value of 24 mm. In contrast, compound **3h**, in which positions 5 and 6 of the oxindole ring are unsubstituted, showed no activity versus *MRSA*. Compounds **3a**–**3g** generally showed negligible antibacterial effects. These findings suggest that the substitution at position 5 or 6 of the oxindole ring with an electron-withdrawing group such as chlorine, together with a para-substituted electron-donating group on the phenacyl moiety, enhances activity against *S. aureus* and *MRSA*.

#### Screening of antifungal activity

As shown in Table 1, ten compounds (**3a–3j**) were assessed against *Candida albicans* and *Aspergillus spp*. using the cup-plate agar diffusion method. Itraconazole served as the antifungal reference drug. The final concentration applied per well was standardized to 100 µg/well for both the test compounds and reference standards (9 mm well diameter)^27^. Eight compounds (**3a-f**, **3i**, and **3j**) inhibited the growth of *Aspergillus spp*. but were inactive against *C. albicans*. The only exception was compound **3g**, which displayed moderate activity against *C. albicans* (IZD = 12 mm). Compound **3f** showed the largest inhibition zone diameter (IZD = 20 mm), followed by compounds **3c** and **3d** (IZD = 17 mm), and compound **3a** (IZD = 16 mm). Both compounds **3b** and **3e** showed an IZD of 15 mm. Antifungal drugs have several mechanisms of action. The most common targets of antifungal drugs are to disrupt cell membranes, cell walls, proteins, and ergosterol biosynthesis^28^. Minor structural differences have been detected between different fungal genera such as *Candida*, *Aspergillus,* and *Cryptococcus*^29,30^. The infections caused by *C. albicans* or *Aspergillus* spp. are treated by three classes of antifungal agents. The first class is the polyene, which acts on the fungal cell membrane. The second class is the azole that inhibits ergosterol biosynthesis, and the last class acts on fungal cell walls, such as echinocandins^30^. Previous studies have illustrated the importance of Nucleoside Diphosphate Kinase (NDK) as an essential enzyme for *Aspergillus* survival, hyphal growth, and conidia production^28,31^. The former observations could explain the higher activity of these compounds against *Aspergillus* spp. than against *C. albicans* due to the possible effect of these compounds on *Aspergillus* NDK.

Consequently, an in-depth investigation is required to enrich our knowledge of the impact of these newly synthesized compounds on fungal growth. Furthermore, it was found that all tested compounds showed no antifungal activity against *Candida albicans* except compound **3g** displayed good antifungal activity compared to the positive control antifungal agent Itraconazole, with inhibition zones’ diameters of 12 mm and 28 mm, respectively. Consequently, the presence of electron-withdrawing groups such as a Br atom at the para position of phenacyl bromide in addition to making position 6 of the oxindole ring substituted with electron electron-withdrawing group such as a Cl atom, like compound **3g** is essential to exhibit acceptable antifungal activity against *Candida albicans.* Though all tested compounds afforded antifungal activity against *Aspergillus spp,* except compounds **3g** and **3h**. In relation to this, compound **3f** revealed higher antifungal activity against *Aspergillus spp* than the tested antifungal drug, promising antifungal activity compared to Itraconazole with inhibition zones’ diameters of 20 mm and 30 mm, respectively.

The structure–activity relationship suggests that *para*-substitution with an electron-withdrawing group (e.g., bromine in **3f**) enhances antifungal activity against *Aspergillus spp*., whereas *para*-methoxy substitution (electron-donating group) (compounds **3h–3j**) reduces activity.

##### MIC determination of 3-(4-(2-(4-bromophenyl)-2-oxoethoxy)benzylidene)indolin-2-one (3f) against *Aspergillus niger*

Due to their prevalence in physiologically active natural and synthetic drugs, indolin-2-one scaffolds (oxindoles) are well-known medicinal science pharmacophores. These compounds have many pharmacological effects, such as antiproliferative, antibacterial, α-glucosidase inhibitory, antileishmanial, antioxidant, tyrosinase inhibitory, and anti-rheumatoid arthritis. Oxindole derivatives functionalized at the C-3 position of the indolin-2-one nucleus are potent kinase inhibitors, as shown by sunitinib (SU11248, SutentTM; Pfizer, Cairo, Egypt) and several other clinical trial candidates^13^. New oxindole compounds with side chains were tested on toxinogenic, phytopathogenic, and dermatophytic filamentous fungi. Higher antifungal activity was seen in derivatives having an exocyclic C = C bond at position C-3 compared to those with a C−C bond. The most effective antifungal agent was 3-(-2-thienoylmethylidene)-indol-2(3*H*)-one^14^.

Minimum inhibitory concentration (MIC) values were determined for compound **3f** against *Aspergillus niger* using the microbroth dilution method. Compound **3f** exhibited an MIC of 7.5 µg/mL, which was lower than that of clotrimazole (12.5 µg/mL). To avoid confusion, itraconazole was used for the diffusion assay comparison, while clotrimazole was employed in MIC testing. Direct comparisons are therefore restricted within each method.

##### Sorbitol assay and ergosterol binding test:

Many antifungal drugs inhibit the growth of fungi by binding ergosterol in the membrane. So, there was a need to verify the effect of the tested drug in the presence of exogenous ergosterol. Decreasing the binding of the tested drugs to the ergosterol of the membrane increases the drug MIC. Sorbitol acts as an osmotic protective agent for fungal cell walls. So, increasing MICs in the presence of sorbitol indicates the activity of the tested compounds on the cell wall. The antifungal activity of the tested drug should be evaluated to determine the possible mechanism of action. It is important to be sure that their effect is due to their action on one of the desired targets, such as the fungal cell wall or the fungal cell membrane, without affecting human cells. So, the tested drug was tested in the presence of ergosterol and sorbitol.

The potential mechanism of antifungal action of **3f** was investigated using ergosterol binding and sorbitol protection assays. The MIC of **3f** remained unchanged in the presence of exogenous ergosterol, whereas clotrimazole showed an eightfold increase, indicating that **3f** does not act through ergosterol binding. In contrast, the MIC of **3f** increased fourfold in the presence of sorbitol, suggesting disruption of fungal cell wall integrity as a likely mechanism.

Studies suggested that these compounds that block kinases could also be effective antifungals^15^. Kinases are essential to microbial growth and survival. 3-substituted benzylideneindolin-2-one derivatives are antibacterial^16,17^, but their antifungal potential is understudied, with most studies focusing on *Candida species*. This is an essential research gap, especially given the rise of fungal infections and medication resistance. A previous study found that 3-alkenyl oxindole derivatives had significant antifungal activity against pathogenic Candida species, suggesting their potential as antifungals^18^.

### Docking studies

To further explore the antifungal mechanism of **3f**, molecular docking studies were conducted against two validated fungal targets: **chitin deacetylase (AngCDA, PDB ID: 7BLY)** and **1,3-β-glucan synthase (PDB ID: 8JZN)**. These enzymes are essential for fungal cell wall biosynthesis and are absent in human cells, making them attractive to therapeutic targets.

Fungi need cell walls for viability, morphogenesis, and pathogenicity^19^. Fungal cell barriers are intriguing targets for antifungal drug research since human cells lack them^20^. Plant cell walls are predominantly constituted of cellulose (1,4-β-glucan), while fungal cell walls are primarily made of 1,3-β-glucan, 1,6-β-glucan, chitin, and mannoproteins^21^. Most approved and emerging antifungals inhibit GS to target cell wall function. This enzyme uses UDP-Glc as a sugar source and transports 1,3-β-glucan through the membrane to create β (1 → 3) glycosidic bonds. Over the past 40 years, only echinocandins and triterpenoids have been launched as novel antifungals. These two types prevent fungal cell wall production by inhibiting 1,3-β-glucan synthase (GS) in the plasma membrane^22^.

**Fig. 4:**
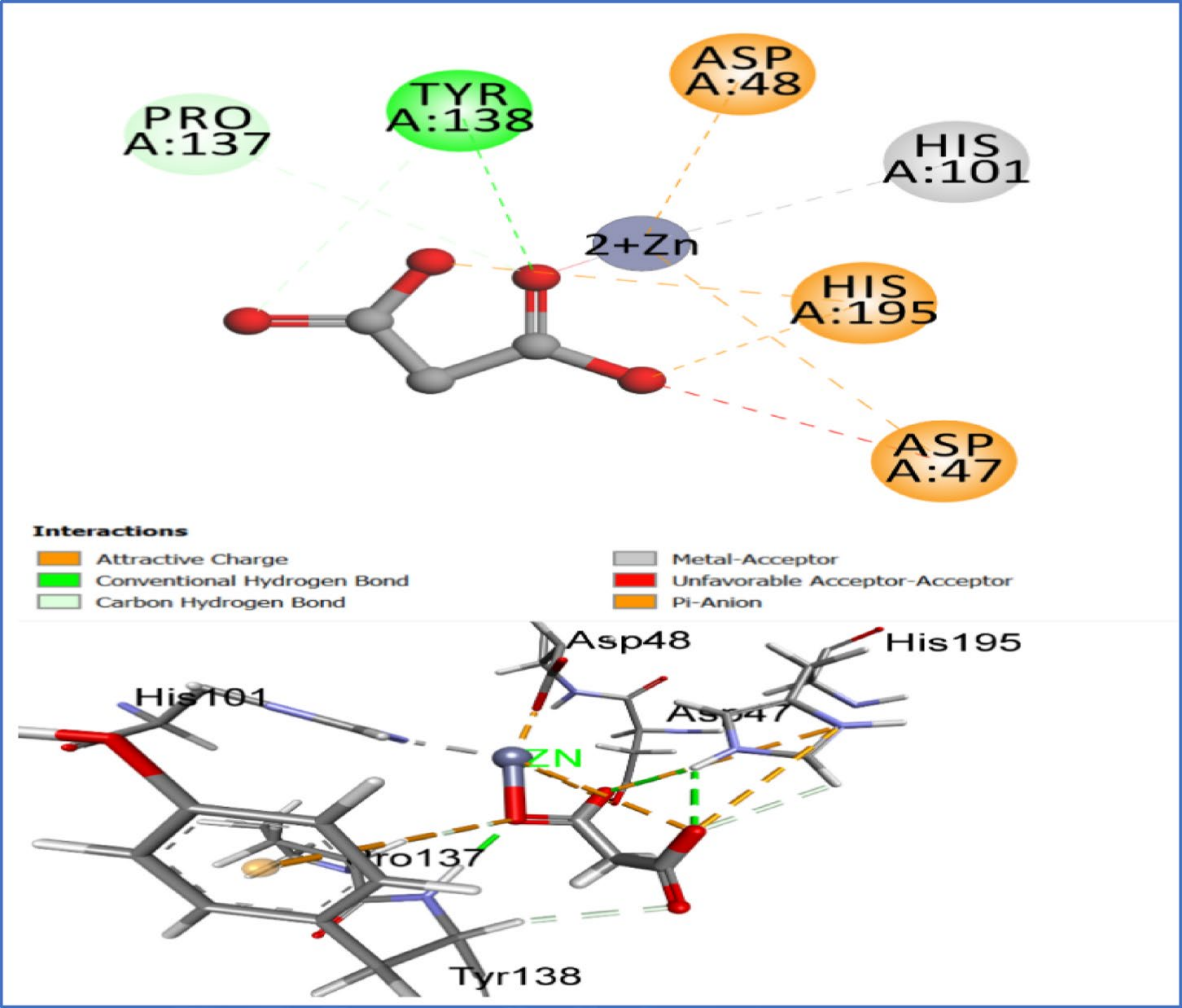
2D and 3D interaction diagrams of the co-crystallized malonate ion within the active site of chitin deacetylase AngCDA (PDB ID: 7BLY). Image created with BIOVIA Discovery Studio Visualizer, v24.1 (Dassault Systèmes, San Diego, CA, USA; https://discover.3ds.com/discovery-studio-visualizer-download?utm_source).

Chitosan, a β 1,4-linked glucosamine polymer, contributes to fungal cell wall function, including virulence and immunological evasion. The subsite capping concept states that these loops block sections of the binding site, causing the substrate to bind in a certain place and preventing deacetylation of produced polymeric substrates. Active CDAs like the fungal AngCDA, ClCDA, and AnCDA have smaller loops that don’t block the binding site, making it more accessible to the substrate. However, their smaller loops dominate the binding site and are likely responsible for these CDAs’ different modalities of activity^23^. To validate the reliability of the molecular docking protocol, a redocking experiment was conducted using the co-crystallized ligand (malonate ion) from the crystal structure of chitin deacetylase. The ligand was extracted, redocked into the active site, and the resulting pose was compared to the experimentally determined structure. The root-mean-square deviation (RMSD) between the docked and crystallographic poses was calculated to be 1.02 Å, indicating a high degree of structural congruence and confirming the accuracy and robustness of the docking parameters. The co-crystallized ligand yielded a binding affinity of − 8.33 kcal/mol, serving as a reference benchmark for evaluating the binding efficiency of the test compounds. The docking results were compared to the native co-crystallized ligand, malonate, which is observed in the crystal structure occupying the catalytic core. The malonate ion displayed classical coordination geometry with the Zn^2^⁺ cofactor, forming bidentate metal-acceptor interactions via its carboxylate groups (Fig. 4). Additionally, it established hydrogen bonds with key residues, including Tyr138 and Pro137, while electrostatic and π-anion interactions were observed with Asp47, Asp48, and His195. These residues form the conserved catalytic triad of CDAs, and the positioning of malonate highlights its role in mimicking a negatively charged substrate intermediate or product state within the catalytic cleft.

**Fig. 5:**
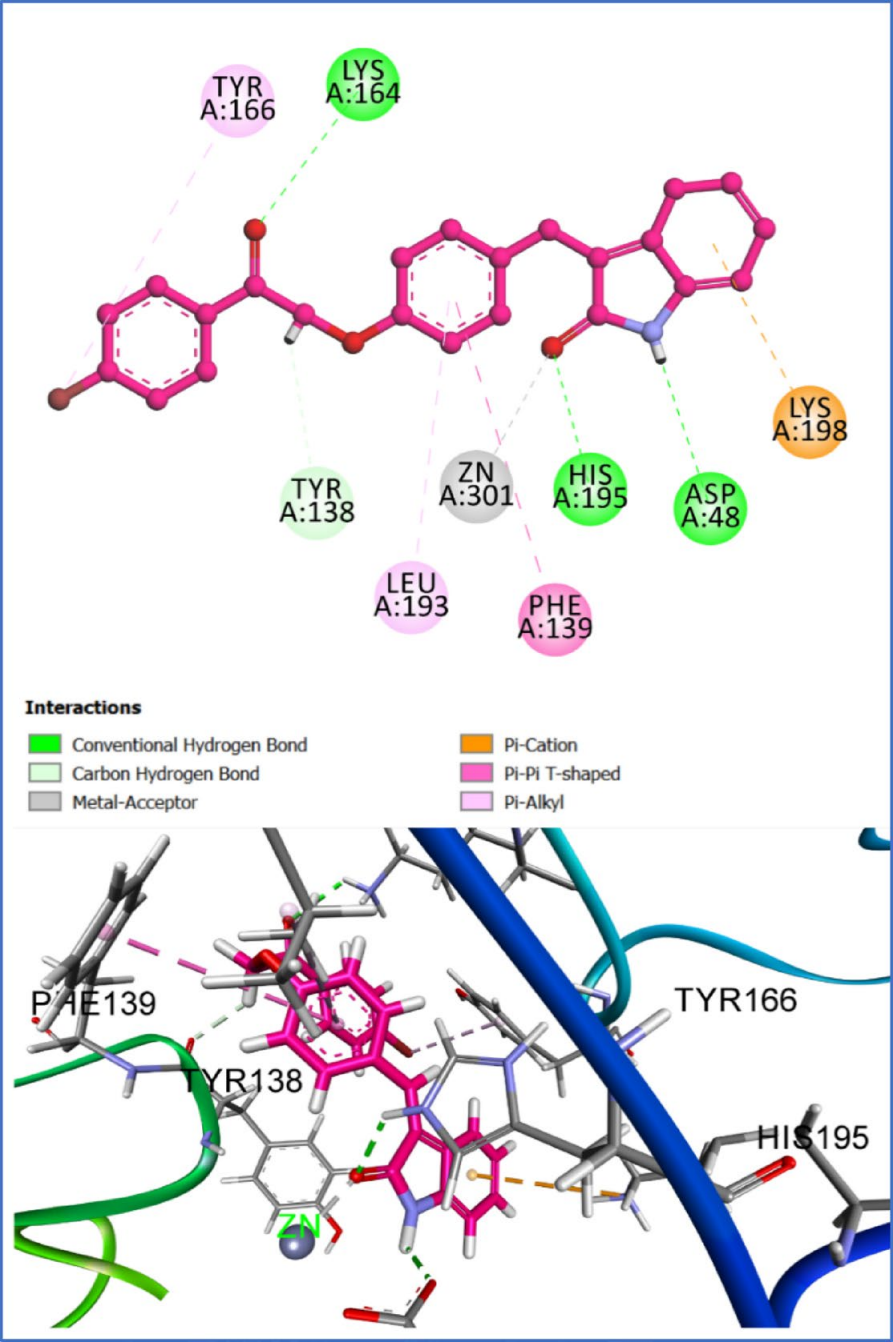
2D and 3D interaction diagrams of compound 3f docked into the active site of chitin deacetylase AngCDA (PDB ID: 7BLY). Image created with BIOVIA Discovery Studio Visualizer, v24.1 (Dassault Systèmes, San Diego, CA, USA; https://discover.3ds.com/discovery-studio-visualizer-download?utm_source).

In comparison, compound **3f** exhibited a predicted binding energy of − 7.33 kcal/mol and an RMSD of 1.42 Å, suggesting a stable and reproducible binding mode within the active site. Docking of compound **3f** revealed a similar Zn^2^⁺ coordination pattern, with one of its carbonyl oxygen atoms directly interacting with the metal ion. The ligand also established key hydrogen bonding interactions with Asp48 and His195, mirroring those of the malonate ion. Importantly, 3f engaged in additional π–π stacking and hydrophobic interactions with aromatic and non-polar residues (Fig. 5), notably Phe139 and Tyr138, enhancing its binding affinity and suggesting a more extensive occupancy of the active site. These interactions suggest that **3f** can mimic not only the electrostatic features of the malonate ion but also extend deeper into the binding pocket, forming stabilizing contacts that are absent in the native ligand. Although slightly less favorable in terms of binding energy, the compound was able to mimic the key coordination and hydrogen bonding interactions observed with the native ligand.

These results support AngCDA inhibition as a plausible mechanism of antifungal action for compound **3f** and demonstrate its potential as a novel lead scaffold for antifungal drug development targeting fungal chitin metabolism.

In addition to targeting chitin deacetylase, molecular docking studies were also performed against 1,3-β-glucan synthase (PDB ID: 8JZN), a key enzyme in fungal cell wall biosynthesis responsible for the polymerization of β-(1 → 3)-glucan chains^24^. This enzyme plays a pivotal role in maintaining fungal cell wall integrity, making it an established and clinically relevant antifungal target, especially in the context of echinocandin and triterpenoid drug classes^25,26^.

To assess the reliability of the docking protocol employed for 1,3-β-glucan synthase (PDB ID: 8JZN), a redocking experiment was performed using the co-crystallized ergosterol analog. The ligand was extracted from the crystal structure and subsequently redocked into its native binding pocket using the same docking parameters applied for test compounds. The resulting docked pose achieved a binding free energy of − 6.84 kcal/mol and an RMSD of 1.71 Å when compared to the crystallographic pose, which falls within the acceptable threshold (< 2.0 Å) for docking accuracy. This RMSD value indicates that the redocking protocol successfully reproduced the native orientation of ligand, validating the docking parameters and search space used in the study.

**Fig. 6:**
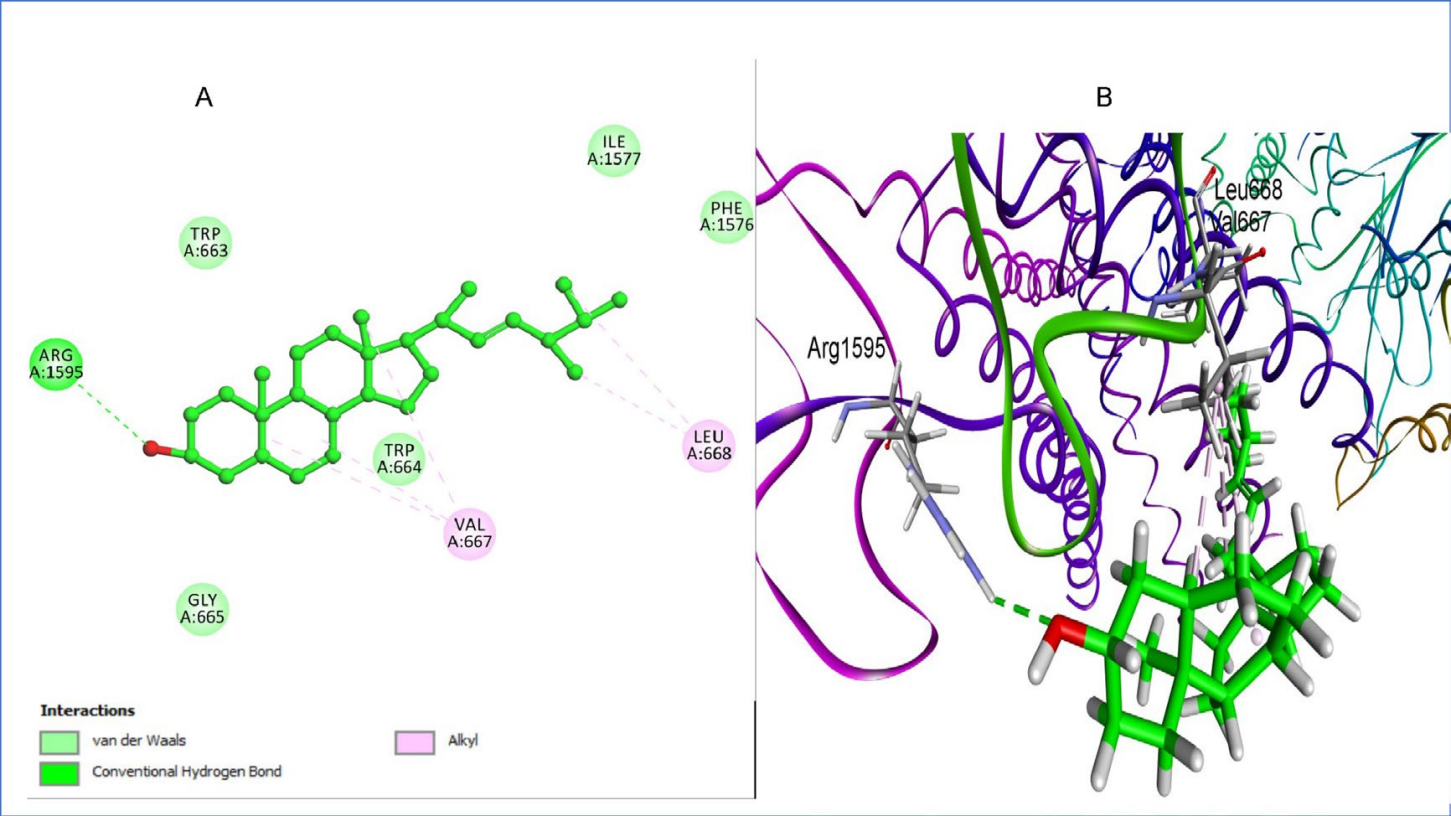
(**A**) 2D interaction diagram of ergosterol within the 1,3-β-glucan synthase binding site, (**B**) 3D representation of ergosterol within the binding pocket. Image created with BIOVIA Discovery Studio Visualizer, v24.1 (Dassault Systèmes, San Diego, CA, USA; https://discover.3ds.com/discovery-studio-visualizer-download?utm_source).

The ergosterol co-ligand demonstrated key interactions within the transmembrane and catalytic core of the enzyme, including hydrophobic contacts with Val667, and Leu668, as well as a hydrogen bond with Arg1595, a residue positioned near the binding cleft (Fig. 6)^27^. These interactions reaffirm the suitability of the selected binding site and the reliability of the docking model for subsequent evaluation of antifungal compounds targeting 1,3-β-glucan synthase.

The docking analysis of fluconazole, a well-known antifungal agent targeting 1,3-β-glucan synthase, provides valuable insights into its binding mode within the active site^28^. Fluconazole exhibits a favorable binding energy of − 7.48 kcal/mol with an RMSD value of 1.21 Å, indicating a stable pose within the 1,3-β-glucan synthase binding pocket. The analysis reveals a conventional hydrogen bond interaction between fluconazole and Arg1595, which plays a crucial role in anchoring the ligand within the binding site. Additionally, pi-cation interactions are observed between triazole ring of fluconazole and Arg1595, further enhancing its stability. The ligand also forms carbon-hydrogen bonds with Trp664, while pi-alkyl interactions with Val667 contribute to a stable hydrophobic environment.

**Fig. 7:**
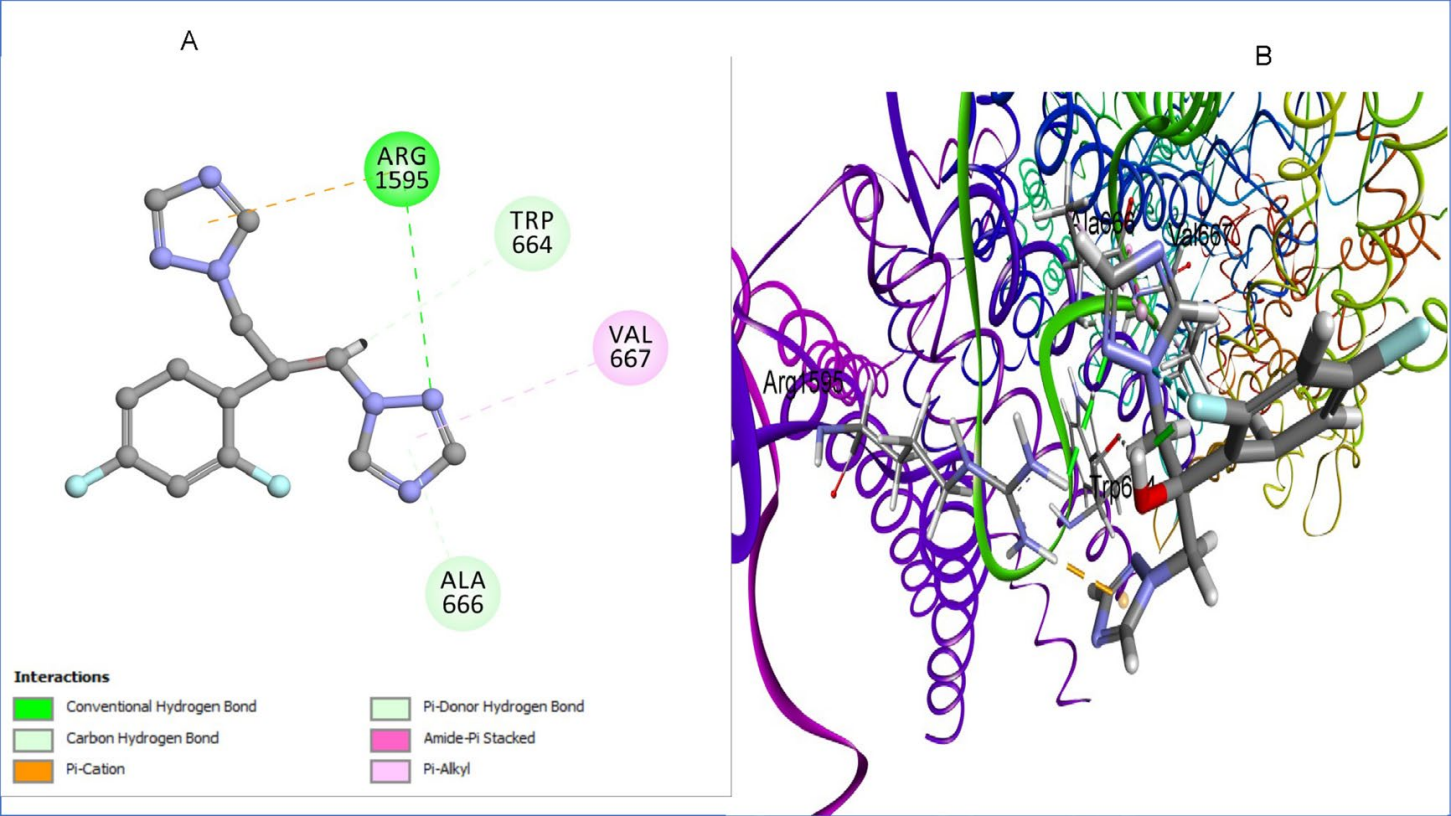
(**A**) 2D interaction diagram of fluconazole within the 1,3-β-glucan synthase binding site, (**B**) 3D representation of fluconazole docked within the 1,3-β-glucan synthase active site. Image created with BIOVIA Discovery Studio Visualizer, v24.1 (Dassault Systèmes, San Diego, CA, USA; https://discover.3ds.com/discovery-studio-visualizer-download?utm_source).

These extensive hydrogen bonding and hydrophobic interactions ensure a strong binding affinity of fluconazole within the 1,3-β-glucan synthase active site, reinforcing its inhibitory potential against the enzyme (Fig. 7).

**Fig. 8:**
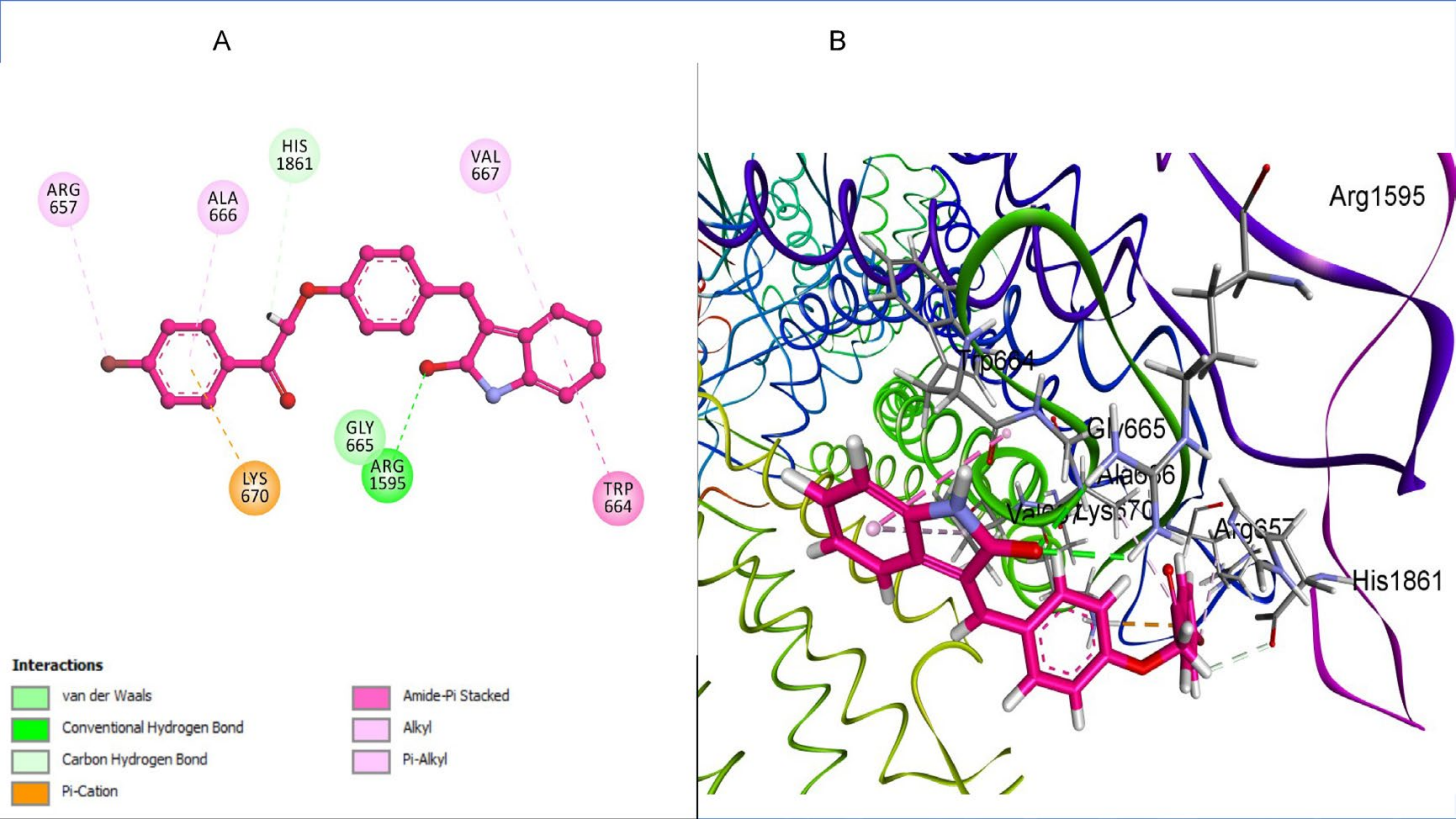
(**A**) 2D interaction diagram of compound 3f within the 1,3-β-glucan synthase binding site, (**B**) 3D representation of compound 3f within the binding pocket of 1,3-β-glucan synthase. Image created with BIOVIA Discovery Studio Visualizer, v24.1 (Dassault Systèmes, San Diego, CA, USA; https://discover.3ds.com/discovery-studio-visualizer-download?utm_source).

The docking analysis of compound **3f** within 1,3-β-glucan synthase revealed a highly favorable binding pose with a docking score of − 7.16 kcal/mol and a root mean square deviation (RMSD) value of 0.96 Å, indicating a stable and well-aligned interaction with the active site. The interaction profile demonstrates that **3f** forms a conventional hydrogen bond with Arg1595, which plays a crucial role in anchoring the ligand. Additionally, carbon-hydrogen bonding with His1861 contributes to further stabilization within the active site. A pi-cation interaction with Lys670 enhances electrostatic complementarity, while pi-alkyl interactions with Val667, and Ala666 provide additional hydrophobic stabilization. The presence of amide-pi stacking interactions with Trp664 further strengthens the ligand binding, suggesting a high affinity of compound 3f toward 1,3-β-glucan synthase, making it a potential inhibitor candidate (Fig. 8).

The molecular docking analyses conducted in this study revealed that the synthesized oxindole derivative compound **3f** exhibits strong and stable binding interactions with two essential fungal enzymes: chitin deacetylase (AngCDA) and 1,3-β-glucan synthase, both of which are critical for maintaining fungal cell wall integrity. Redocking experiments validated the reliability of the docking protocols, with RMSD values within acceptable thresholds (< 2.0 Å). Compound **3f** demonstrated favorable binding affinities (− 7.33 and − 7.16 kcal/mol, respectively), comparable to or better than those of the co-crystallized ligands and the marketed antifungal fluconazole. Key interactions included Zn^2^⁺ coordination, hydrogen bonding, and hydrophobic interactions with catalytically important residues in both targets. Notably, compound **3f** was able to mimic the binding profile of native ligands while offering enhanced engagement through extended hydrophobic and π-stacking interactions. These findings suggest that compound **3f** is a promising dual-target antifungal candidate capable of disrupting fungal cell wall biosynthesis through simultaneous inhibition of chitin deacetylation and β-glucan polymerization, warranting further experimental validation and optimization in future studies.

## Conclusion

The compounds **3i** and **3j** showed higher antibacterial activity than other tested compounds. Eight compounds (**3a**-**f**, **3i,** and **3j**) exhibited remarkable antifungal activity against *Aspergillus* spp. and no effect on *C. albicans*.

Compound **3f** revealed promising antifungal activity against *Aspergillus spp*, compared to the tested antifungals. These results identify compound **3f** as a promising scaffold for the development of novel antifungal agents targeting fungal cell wall biosynthesis.

## Materials and methods

### Chemistry

#### General procedures for the synthesis of target compounds 3a-j

A mixture of compounds **1a-c** (1 mmol) and 2-oxindole or oxindole derivatives **2a-e** (1 mmol) in absolute ethanol (50 mL) and piperidine (85 mg, 1 mmol) was refluxed for 24 h. Compounds **3a-j** were formed on hot, filtrated, washed several times with hot ethanol, and dried in a vacuum oven to afford compounds **3a-j**^18^.

### Biological investigation

The antifungals Fluconazole and Ergosterol were obtained from Sigma-Aldrich® (São Paulo, São Paulo, Brazil). The sorbitol was obtained from INLAB® (São Paulo, Brazil). Muller-Hinton agar was obtained from LabM, DMSO (dimethylsulfoxide – Sigma-Aldrich®, São Paulo, São Paulo, Brazil).

#### Screening of antimicrobial activity of compounds 3a-j

##### Screening of antibacterial activity

The antibacterial Screening was performed by the **cup plate agar diffusion method**^19^ against *S. aureus* (ATCC29213), *Enterococcus faecalis,* multidrug resistance (MDR) isolates such as Methicillin-Resistant *Staphylococcus aureus* (*MRSA*), and Gram-negative bacteria *E. coli* (ATCC8729), *Klebsiella pneumonia*, and *Pseudomonas aeruginosa*. The antibacterial activity is detected as follows: a hole with a diameter of 9 mm is punched aseptically into a seeded Mueller–Hinton (MH) agar plate with a sterile cork borer or a tip, and a volume (100 µL) of each compound at the desired concentration (10,000 μg/mL) is introduced into the well. Ciprofloxacin will be used as the reference antibiotic (100 μg/mL). The inhibition zone was measured after 24 h of incubation at 37 °C.

##### Screening of antifungal activity

The antifungal Screening was carried out using the cup plate agar diffusion method against *Candida albicans* and *Aspergillus spp*. The antifungal activity is detected as the following procedures; a hole with a diameter of 9 mm is punched aseptically into a seeded Mueller–Hinton (MH) agar (supplemented with 2% glucose) plates with a sterile cork borer or a tip, and a volume (100 µL) of each compound at desired concentration (5000 & 10,000 μg/mL) is introduced into the well. Itraconazole (100 μg/mL) will be served as a reference antifungal, and DMSO as the control. The zone of inhibition was measured after 24–48 h of incubation at 30 °C.

#### Minimum inhibitory concentration determination using the broth dilution method:

The MIC of (Z)-3-(4-(2-(4-bromophenyl)-2-oxoethoxy)benzylidene)indolin-2-one and antifungal drug were determined using spore suspension (1.5 × 10^5^ CFU mL − 1). One hundred microliter of the tested compound and clotrimazole at concentrations ranging from 1024 to 2.5 μg/mL (two-fold serial dilutions) were distributed in 96-well “U” plates^32^. Ten microliters of spore suspension was added to each well. Plates were incubated at 30°C for 3 days. MIC was the lowest concentration that prevented visible growth^29,30^.

#### Sorbitol assay effects

To determine how the test product inhibited *Aspergillus spp.* cell walls, the assay was done in medium with and without sorbitol (control). Sorbitol was added to the culture medium at 0.8 M. Microdilution was used in 96-well “U” plates for the test. Aseptically sealed plates were incubated at 28 °C and measured after 3 days. Sorbitol acts as a fungal cell wall osmotic protecting agent; hence, the higher MIC values in the medium with added sorbitol than in the standard medium suggest the cell wall as a likely cell target for the product examined. Control medication was amphotericin B. The geometric mean of the duplicate assay was reported^31^.

#### Ergosterol binding assay

Ergosterol MIC Value Determination. A modified Escalante et al.^31^ experiment was used to determine if the product binds to fungal membrane sterols. Ergosterol was produced as described by Leite et al.^32^. The MIC of (Z)-3-(4-(2-(4-bromophenyl)-2-oxoethoxy)benzylidene)indolin-2-one against *Aspergillus spp.* was determined using broth microdilution techniques^33,34^. The assay was conducted with and without exogenous ergosterol (Sigma-Aldrich, Sao Paulo, ˜ SP, Brazil) in different microplate lines. A solution of (Z)-3-(4-(2-(4-bromophenyl)-2-oxoethoxy) benzylidene) indolin-2-one was serially diluted with 400 μg/mL ergosterol. A 10 μL fungal suspension (0.5 McFarland) was applied to each well. As a control medication, Amphotericin B, which interacts with membrane ergosterol, was treated similarly. The plates were sealed and incubated at 28°C. After 3 days of incubation, the plates were examined to determine the MIC, the lowest test agent concentration preventing observable growth. In duplicate, the test was done, and the geometric mean.

### Molecular docking

The chemical structures of every synthesized molecule were designed in ChemDraw Ultra 8.0, and Marvin Sketch was used to modify all derivative Mol2 files.pdb files during ligand production. After becoming flexible, each ligand molecule’s torsional roots were selected. PDB files were optimized and converted to PDBQT files for molecular docking using AutoDock Tools 1.5.6. The Protein DataBank was utilized to find the crystal structures of Aspergillus niger’s 1,3-beta-glucan synthase (8JZN) antifungal target protein and chitin deacetylase AngCDA (7BLY).

A target molecule in *.pdb format was downloaded, ligands were removed, polar hydrogen atoms were inserted, and bond ordering were set to create protein structure. The macromolecule’s *.pdb format must be converted to *.pdbqt. AutoDock Tools was used to generate autogrids and determine x, y, and z coordinates. Grid-based cavity prediction was used to find the binding site. Molecular docking was carried out in AutoDock Vina using default parameters: exhaustiveness = 8, energy range = 3 kcal/mol, and num_modes = 8, to evaluate a synthetic series of drugs against antifungal targets^35,36^. The cnf.txt file was created containing a grid center, x, y, and z coordinates in Angstrom, a grid box size in Å, and the receptor and ligand in.pdbqt format. The main conformers were identified using the Lamarckian Genetic Algorithm (LGA). The photos of ligand-receptor interactions showed the kind, distance, and ligand and protein atoms involved. It also locates ligand-engaged receptor active functional sites. Assessments used free binding energy and hydrogen bonding. An inhibitory effect of ligands is assessed by docking analysis using the principle that a lower protein binding free energy means a higher ligand binding capacity. To verify that the synthesized derivatives were linked to the drug targets’ active sites, PyMol Molecular Graphics System was used to build receptor-ligand complexes.

## Supplementary Information

Below is the link to the electronic supplementary material.

Supplementary Material 1

## Data Availability

All data generated or analyzed during this study are included in this published article [and its supplementary information files].
