# Supplementary material for: Antimicrobial activity and antifungal mechanistic study of 3‑substituted oxindoles against Aspergillus niger
Source: Sci Rep. 2025 Nov 29;15:42918. doi: 10.1038/s41598-025-27782-4 (PMC12672562; doi:10.1038/s41598-025-27782-4)
Supplement: Supplementary file 1 — Supplementary Material 1 [file 41598_2025_27782_MOESM1_ESM.docx]

**Antimicrobial activity and antifungal mechanistic study of 3‑substituted oxindoles against** ***Aspergillus niger***

**Hend A. A.** [**Ezelarab**](https://www.researchgate.net/profile/Hend_Ezelarab?_sg%5B0%5D=mkPHDtm-YANKygOyzayqFrjB3TeL8vEHhPoI5d0hRegJDi18-96m7mB75kJExYHROIl0Qk0.FFpfqe6owJHXeo6u7rcAFPhhI2_xdllgqU8WgE-d_34UjHOBsc1Wu3WgrY2Gksn08gkHDUzt7bPn4TpO2PPV2Q&_sg%5B1%5D=VhRrT7PATBq1bQisAs0zLLUBQrQxkYSNJvp_QHkFnmtDTVACy61srBT9c5r0Sdp7PSICZR4pb4CQHWPs.UOmMD95eiSa04RnTM70npebwuV9Ml-hMcWezDC2uJLxfT47d2vVC_XmKF2RG4vNAG5Sd1IAg81JzwzxV46SM-g)**^1,,2, #^,** **Maisra M. El-Bouseary^3,#^, Ramadan Yahia^4^, Rehab Mahmoud Abd El-Baky^5,6,*^, Mohamed A. Mawhoup^6^, Eman Farouk Ahmed^7^, Ghada M. Sadiq^8^, Taha F. S. Ali^1,2^,** **[Samar H Abbas](https://www.researchgate.net/profile/Samar_Abbas6?_sg%5B0%5D=mkPHDtm-YANKygOyzayqFrjB3TeL8vEHhPoI5d0hRegJDi18-96m7mB75kJExYHROIl0Qk0.FFpfqe6owJHXeo6u7rcAFPhhI2_xdllgqU8WgE-d_34UjHOBsc1Wu3WgrY2Gksn08gkHDUzt7bPn4TpO2PPV2Q&_sg%5B1%5D=VhRrT7PATBq1bQisAs0zLLUBQrQxkYSNJvp_QHkFnmtDTVACy61srBT9c5r0Sdp7PSICZR4pb4CQHWPs.UOmMD95eiSa04RnTM70npebwuV9Ml-hMcWezDC2uJLxfT47d2vVC_XmKF2RG4vNAG5Sd1IAg81JzwzxV46SM-g)^1,2,*^,** [**Heba A Hassan**](https://www.researchgate.net/profile/Heba_Hassan30?_sg%5B0%5D=mkPHDtm-YANKygOyzayqFrjB3TeL8vEHhPoI5d0hRegJDi18-96m7mB75kJExYHROIl0Qk0.FFpfqe6owJHXeo6u7rcAFPhhI2_xdllgqU8WgE-d_34UjHOBsc1Wu3WgrY2Gksn08gkHDUzt7bPn4TpO2PPV2Q&_sg%5B1%5D=VhRrT7PATBq1bQisAs0zLLUBQrQxkYSNJvp_QHkFnmtDTVACy61srBT9c5r0Sdp7PSICZR4pb4CQHWPs.UOmMD95eiSa04RnTM70npebwuV9Ml-hMcWezDC2uJLxfT47d2vVC_XmKF2RG4vNAG5Sd1IAg81JzwzxV46SM-g)**^1,9^,** **Eman A M Beshr^1,2,*^.**

^1^Department of Medicinal Chemistry, Faculty of Pharmacy, Minia University, 61519-Minia, Egypt.

^2^Medicinal Chemistry Department, Faculty of Pharmacy, Minia National University, New Minia, Egypt

^3^Department of Pharmaceutical Microbiology, Faculty of Pharmacy, Tanta University,

^4^Microbiology and Immunology Department, Faculty of Pharmacy, Badr University in Assiut, Assiut ‎‎77771, Egypt.

^5^ Microbiology and Immunology Department, Faculty of Pharmacy, Minia University, 61519, Minia, Egypt.

^6^ Microbiology and Immunology department, faculty of pharmacy, Deraya University, Minia, Egypt.

^7^Department of Microbiology, Faculty of Pharmacy, Sohag University, Sohag, Egypt.

^8^Department of Pharmaceutical Chemistry, Faculty of Pharmacy, Deraya University.

^9^Pharmaceutical Chemistry Department, Faculty of Pharmacy, Badr University in Assiut

(BUA), Assiut 2014101, Egypt

***** Corresponding Author

Rehab Mahmoud Abd El-Baky; E-mail: [rehab.mahmoud@mu.edu.eg](mailto:rehab.mahmoud@mu.edu.eg)

Eman A M Beshr; E-mail: [eman_beshr@mu.edu.eg](mailto:eman_beshr@mu.edu.eg)

[Samar H Abbas](https://www.researchgate.net/profile/Samar_Abbas6?_sg%5B0%5D=mkPHDtm-YANKygOyzayqFrjB3TeL8vEHhPoI5d0hRegJDi18-96m7mB75kJExYHROIl0Qk0.FFpfqe6owJHXeo6u7rcAFPhhI2_xdllgqU8WgE-d_34UjHOBsc1Wu3WgrY2Gksn08gkHDUzt7bPn4TpO2PPV2Q&_sg%5B1%5D=VhRrT7PATBq1bQisAs0zLLUBQrQxkYSNJvp_QHkFnmtDTVACy61srBT9c5r0Sdp7PSICZR4pb4CQHWPs.UOmMD95eiSa04RnTM70npebwuV9Ml-hMcWezDC2uJLxfT47d2vVC_XmKF2RG4vNAG5Sd1IAg81JzwzxV46SM-g); E-mail: [samar_hafez@mu.edu.eg](mailto:samar_hafez@mu.edu.eg)

**Methods:**

**1.Tested Microorganisms:**

- Reference strains

- Gram positive bacteria **S. aureus** (**ATCC29213**)
- Gram negative bacteria ***E. coli*** (**ATCC8729**).

- Multidrug resistance (MDR) isolates

- Methicillin Resistant *Staphylococcus aureus* (**MRSA**)
- **Enterococcus faecalis**
- **Klebsiella pneumonia*e***,
- **Pseudomonas aeruginosa**,

- Fungi

- ***Candida albicans***
- ***Aspergillus spp***.

**2. Screening of antimicrobial activity:**

The antibacterial screening was performed by **cup plate** **agar** **diffusion method** against the selected isolates.

**Antibacterial activity**: A hole with a diameter of ~ 9 mm is punched aseptically into a seeded Mueller-Hinton (MH) agar plates with a sterile cork borer or a tip, and a volume (20–100 µL) of each compound at desired concentration (5000 & 10000 μg/mL) is introduced into the well. Ciprofloxacin, Streptomycin sulfate and oxindole will be served as reference antibiotics and DMSO as the control. Zone of inhibition was measured after 24 h of incubation at 37°C.

**Antifungal activity**: A hole with a diameter of ~ 9 mm is punched aseptically into a seeded Mueller-Hinton (MH) agar (supplemented with 2% glucose) plates with a sterile cork borer or a tip, and a volume (20–100 µL) of each compound at desired concentration (5000 & 10000 μg/mL) is introduced into the well. Itraconazole will be served as reference antifungal and DMSO as the control. Zone of inhibition was measured after 24-48 h of incubation at 30°C.

**Results:**

1. Effect of tested compounds on ***Staphylococcus aureus* (ATCC29213)**

| Sample Code | Sample ID | 10000 μg/ml | | | | (Inhibition zone in mm)  (Mean± SD) |
| --- | --- | --- | --- | --- | --- | --- |
|  |  | Group 1 | Group 2 | | Group 3 |  |
| 1 | 3a | - | | - | - | - |
| 2 | 3b | - | | - | - | - |
| 3 | 3c | - | | - | - | - |
| 4 | 3d | - | | - | - | - |
| 5 | 3e | - | | - | - | - |
| 6 | 3h | 13 ± 0.3 | | 14 ± 0.3 | 13 ± 0.3 | 13 ± 0.3 |
| 7 | 3i | 13 ± 0.3 | | 13 ± 0.3 | 14 ± 0.3 | 13 ± 0.3 |
| 8 | 3j | 16 ± 0.3 | | 15 ± 0.3 | 15 ± 0.3 | 15 ± 0.3 |
| 9 | 3f | - | | - | - | - |
| 10 | 3g | - | | - | - | - |
| CIP | Ciprofloxacin | 30 ± 1.00 | | 28 ± 1.00 | 29 ± 1.00 | 28 ± 1.00 |
| OXI | Oxindole | 15 ± 0.3 | | 14 ± 0.3 | 14 ± 0.3 | 14 ± 0.3 |
| DMSO | DMSO | - | | - | - | - |

(-: means no inhibition zone detected as our zero value is the 9 mm which is the diameter of our hole cup).

Inhibition zone (mm)= Mean ± SD, n = 3.

2. Effect of tested compounds on ***MRSA***

| Sample Code | Sample ID | 10000 μg/ml | | | | Inhibition zone in mm)  (Mean± SD) |
| --- | --- | --- | --- | --- | --- | --- |
|  |  | Group 1 | Group 2 | | Group 3 |  |
| 1 | 3a | - | | - | - | - |
| 2 | 3b | - | | - | - | - |
| 3 | 3c | - | | - | - | - |
| 4 | 3d | - | | - | - | - |
| 5 | 3e | - | | - | - | - |
| 6 | 3h | - | | - | - | - |
| 7 | 3i | 13 ± 0.3 | | 13 ± 0.3 | 14 ± 0.3 | 13 ± 0.3 |
| 8 | 3j | 16 ± 0.3 | | 15 ± 0.3 | 16 ± 0.3 | 16 ± 0.3 |
| 9 | 3f | - | | - | - | - |
| 10 | 3g | - | | - | - | - |
| CIP | Ciprofloxacin | 25 ± 1.00 | | 23 ± 1.00 | 24 ± 1.00 | 24 ± 1.00 |
| OXI | Oxindole | 13 ± 0.3 | | 12 ± 0.3 | 12 ± 0.3 | 12 ± 0.3 |
| DMSO | DMSO | - | | - | - | - |

(-: means no inhibition zone detected as our zero value is the 9 mm which is the diameter of our hole cup).

Inhibition zone (mm)= Mean ± SD, n = 3.

3. Effect of tested compounds on ***Enterococcus faecalis***

| Sample Code | Sample ID | 10000 μg/ml | | | | Inhibition zone in mm)  (Mean± SD) |
| --- | --- | --- | --- | --- | --- | --- |
|  |  | Group 1 | Group 2 | | Group 3 |  |
| 1 | 3a | - | | - | - | - |
| 2 | 3b | - | | - | - | - |
| 3 | 3c | - | | - | - | - |
| 4 | 3d | - | | - | - | - |
| 5 | 3e | - | | - | - | - |
| 6 | 3h | - | | - | - | - |
| 7 | 3i | 15 ± 0.3 | | 14 ± 0.3 | 14 ± 0.3 | 14 ± 0.3 |
| 8 | 3j | - | | - | - | - |
| 9 | 3f | - | | - | - | - |
| 10 | 3g | 12 ± 0.3 | | 12 ± 0.3 | 11 ± 0.3 | 12 ± 0.3 |
| CIP | Ciprofloxacin | 31 ± 1.00 | | 32 ± 1.00 | 33 ± 1.00 | 32 ± 1.00 |
| OXI | Oxindole | 13 ± 0.3 | | 12 ± 0.3 | 12 ± 0.3 | 12 ± 0.3 |
| DMSO | DMSO | - | | - | - | - |

(-: means no inhibition zone detected as our zero value is the 9 mm which is the diameter of our hole cup).

Inhibition zone (mm)= Mean ± SD, n = 3.

4. Effect of tested compounds on ***E. coli (ATCC8729)***

| Sample Code | Sample ID | 10000 μg/ml | | | | Inhibition zone in mm)  (Mean± SD) |
| --- | --- | --- | --- | --- | --- | --- |
|  |  | Group 1 | Group 2 | | Group 3 |  |
| 1 | 3a | - | | - | - | - |
| 2 | 3b | - | | - | - | - |
| 3 | 3c | - | | - | - | - |
| 4 | 3d | - | | - | - | - |
| 5 | 3e | - | | - | - | - |
| 6 | 3h | - | | - | - | - |
| 7 | 3i | - | | - | - | - |
| 8 | 3j | - | | - | - | - |
| 9 | 3f | - | | - | - | - |
| 10 | 3g | - | | - | - | - |
| CIP | Ciprofloxacin | 38 ± 1.00 | | 37 ± 1.00 | 36 ± 1.00 | 37 ± 1.00 |
| OXI | Oxindole | 13 ± 0.3 | | 13 ± 0.3 | 14 ± 0.3 | 13 ± 0.3 |
| DMSO | DMSO | - | | - | - | - |

(-: means no inhibition zone detected as our zero value is the 9 mm which is the diameter of our hole cup).

Inhibition zone (mm)= Mean ± SD, n = 3.

5. Effect of tested compounds on ***Pseudomonas aeruginosa***

| Sample Code | Sample ID | 10000 μg/ml | | | | Inhibition zone in mm)  (Mean± SD) |
| --- | --- | --- | --- | --- | --- | --- |
|  |  | Group 1 | Group 2 | | Group 3 |  |
| 1 | 3a | - | | - | - | - |
| 2 | 3b | - | | - | - | - |
| 3 | 3c | - | | - | - | - |
| 4 | 3d | - | | - | - | - |
| 5 | 3e | - | | - | - | - |
| 6 | 3h | 13 ± 0.3 | | 12 ± 0.3 | 12 ± 0.3 | 12 ± 0.3 |
| 7 | 3i | - | | - | - | - |
| 8 | 3j | 10 ± 0.3 | | 11 ± 0.3 | 11 ± 0.3 | 11 ± 0.3 |
| 9 | 3f | 12 ± 0.3 | | 11 ± 0.3 | 12 ± 0.3 | 12 ± 0.3 |
| 10 | 3g | - | | - | - | - |
| CIP | Ciprofloxacin | 45 ± 1.00 | | 44 ± 1.00 | 43 ± 1.00 | 44 ± 1.00 |
| OXI | Oxindole | 11 ± 0.3 | | 12 ± 0.3 | 12 ± 0.3 | 12 ± 0.3 |
| DMSO | DMSO | - | | - | - | - |

(-: means no inhibition zone detected as our zero value is the 9 mm which is the diameter of our hole cup).

Inhibition zone (mm)= Mean ± SD, n = 3.

6. Effect of tested compounds on ***Klebsiella pneumoniae***

| Sample Code | Sample ID | 10000 μg/ml | | | | Inhibition zone in mm)  (Mean± SD) |
| --- | --- | --- | --- | --- | --- | --- |
|  |  | Group 1 | Group 2 | | Group 3 |  |
| 1 | 3a | - | | - | - | - |
| 2 | 3b | - | | - | - | - |
| 3 | 3c | - | | - | - | - |
| 4 | 3d | - | | - | - | - |
| 5 | 3e | - | | - | - | - |
| 6 | 3h | - | | - | - | - |
| 7 | 3i | - | | - | - | - |
| 8 | 3j | - | | - | - | - |
| 9 | 3f | - | | - | - | - |
| 10 | 3g | - | | - | - | - |
| CIP | Ciprofloxacin | 15 ± 1.00 | | 16 ± 1.00 | 17 ± 1.00 | 16 ± 1.00 |
| OXI | Oxindole | 13 ± 0.3 | | 12 ± 0.3 | 12 ± 0.3 | 12 ± 0.3 |
| DMSO | DMSO | - | | - | - | - |

(-: means no inhibition zone detected as our zero value is the 9 mm which is the diameter of our hole cup).

Inhibition zone (mm)= Mean ± SD, n = 3.

7. Effect of tested compounds on ***Candida albicans***

| Sample Code | Sample ID | 10000 μg/ml | | | | Inhibition zone in mm)  (Mean± SD) |
| --- | --- | --- | --- | --- | --- | --- |
|  |  | Group 1 | Group 2 | | Group 3 |  |
| 1 | 3a | - | | - | - | - |
| 2 | 3b | - | | - | - | - |
| 3 | 3c | - | | - | - | - |
| 4 | 3d | - | | - | - | - |
| 5 | 3e | - | | - | - | - |
| 6 | 3h | - | | - | - | - |
| 7 | 3i | - | | - | - | - |
| 8 | 3j | - | | - | - | - |
| 9 | 3f | - | | - | - | - |
| 10 | 3g | 12 ± 0.3 | | 11 ± 0.3 | 12 ± 0.3 | 12 ± 0.3 |
| Itra | Itraconazole | 29 ± 0.3 | | 28 ± 0.3 | 28 ± 0.3 | 28 ± 0.3 |
| OXI | Oxindole | 16 ± 0.3 | | 16 ± 0.3 | 17 ± 0.3 | 16 ± 0.3 |
| DMSO | DMSO | - | | - | - | - |

(-: means no inhibition zone detected as our zero value is the 9 mm which is the diameter of our hole cup).

Inhibition zone (mm)= Mean ± SD, n = 3.

8. Effect of tested compounds on ***Aspergillus spp.***

| Sample Code | Sample ID | 10000 μg/ml | | | | Inhibition zone in mm)  (Mean± SD) |
| --- | --- | --- | --- | --- | --- | --- |
|  |  | Group 1 | Group 2 | | Group 3 |  |
| 1 | 3a | 15 ± 0.3 | | 16 ± 0.3 | 16 ± 0.3 | 16 ± 0.3 |
| 2 | 3b | 14 ± 0.3 | | 15 ± 0.3 | 15 ± 0.3 | 15 ± 0.3 |
| 3 | 3c | 16 ± 1.00 | | 17 ± 1.00 | 18 ±1.00 | 17 ± 1.00 |
| 4 | 3d | 18± 1.00 | | 17 ± 1.00 | 16± 1.00 | 17 ± 1.00 |
| 5 | 3e | 14± 1.00 | | 15 ± 1.00 | 16 ± 1.00 | 15 ± 1.00 |
| 6 | 3h | - | | - | - | - |
| 7 | 3i | 13 ± 0.3 | | 12 ± 0.3 | 12 ± 0.3 | 12 ± 0.3 |
| 8 | 3j | 15 ± 0.3 | | 14 ± 0.3 | 14 ± 0.3 | 14 ± 0.3 |
| 9 | 3f | 21 ± 0.3 | | 20 ± 0.3 | 20 ± 0.3 | 20 ± 0.3 |
| 10 | 3g | - | | - | - | - |
| Itra | Itraconazole | 29 ± 0.3 | | 30 ± 0.3 | 30 ± 0.3 | 30 ± 0.3 |
| OXI | Oxindole | 16 ± 0.3 | | 17 ± 0.3 | 17 ± 0.3 | 17 ± 0.3 |
| DMSO | DMSO | - | | - | - | - |

(-: means no inhibition zone detected as our zero value is the 9 mm which is the diameter of our hole cup).

Inhibition zone (mm)= Mean ± SD, n = 3.

**MIC determination of 3-(4-(2-(4-bromophenyl)-2-oxoethoxy)benzylidene)indolin-2-one (3f) and other compounds against *Aspergillus niger*.**

| **The tested compounds** | **MIC (µg/ml)** |
| --- | --- |
| **3a** | 75 |
| **3b** | 75 |
| **3c** | 120 |
| **3d** | >120 |
| **3e** | 150 |
| **3f** | 7.5 |
| **3g** | 200 |
| **3h** | >200 |
| **3i** | 300 |
| **3j** | 200 |
| **Clotrimazole** | 12.5 |

**Sorbitol assay and ergosterol binding test:**

| The tested compounds | MIC (µg/ml) | | |
| --- | --- | --- | --- |
|  | alone | With ergosterol | With sorbitol |
| 3f | 7.5 | 7.5 | 30 |
| clotrimazole | 12.5 | 100 | 12.5 |

**General procedures for the synthesis of target compounds** **1a-c**

A mixture of phenacyl bromide (1 mmol), *p*-hydroxy benzaldehyde (122 mg, 1 mmol), and potassium carbonate (276 mg, 2 mmol) in acetonitrile (50 mL) was refluxed for 24 h. The reaction mixture is cooled to room temperature, diluted with water, then extracted three times with ethyl acetate. After drying the organic layer with anhydrous MgSO_4_, the product was obtained as yellow powder *via* evaporation of the ethyl acetate layer. The obtained residue was recrystallized from acetonitrile to afford the target compounds **1a-c**.

**4-(2-Oxo-2-phenylethoxy) benzaldehyde (1a)**

Yellow crystals; 0.11 g, 22.64 % yield; mp: 118ºC; (Reported 120ºC). ^[83,84]^

**4-(2-(4-Bromophenyl)-2-oxoethoxy) benzaldehyde (1b)**

Yellowish white crystals; 0.11 g, 62.3 % yield; mp: 97-99ºC; (Reported 98ºC).^[85]^

**4-(2-(4-Methoxyphenyl)-2-oxoethoxy) benzaldehyde (1c)**

White crystals; 0.11 g, 22.64 % yield; mp: 83-85ºC; (Reported 80-85ºC). ^[83,84]^

**4.1.3. General procedures for the synthesis of target compounds** **3a-j**

A mixture of compounds **4a-c** (1 mmol) and 2-oxindole or oxindole derivatives **5a-e** (1 mmol) in absolute ethanol (50 mL) and piperidine (85 mg, 1mmol) was refluxed for 24 h. Compounds **6a-j** were formed on hot, filtrated, washed several times with hot ethanol, and dried in a vacuum oven to afford compounds **3a-j**.

**3-{4-[2-Oxo-2-phenylethoxy] benzylidene}indolin-2-one (3a)**

Yellow powder; 0.14 g, 31.19 % yield; mp: 219-221ºC; *R*_f_ = 0.33 (Pet.ether/ethyl acetate, 6:2.5); ^1^H NMR (400 MHz, DMSO-*d*_6_) δ 10.55 (s, 1H, N*H*), 8.04 (d, *J* = 8.5 Hz, 2H, Ar*H*), 7.70 (d, *J* = 8.5 Hz, 3H, Ar*H*), 7.65 (d, *J* = 7.8 Hz, 1H, Ar*H*), 7.60 (s, 1H, Ar*H*), 7.58 (d, *J* = 4.5 Hz, 2H, Ar*H*), 7.21 (t, *J* = 8.1 Hz, 1H, Ar*H*), 7.12 (d, *J* = 8.8 Hz, 2H, Ar*H*), 6.89-6.85 (m, 2H, Ar*H*), 5.71 (s, 2H, CO-C*H_2_-*O); ^13^C NMR (100 MHz, DMSO-*d*_6_) δ 194.24, 168.91, 159.27, 142.70, 135.92, 134.31, 133.91, 131.43, 129.76, 128.88, 127.92, 126.99, 125.73, 122.10, 121.14, 114.98, 114.47, 110.07, 70.26; Anal. Calcd. for C_23_H_17_NO_3_ (355.12): C, 77.73; H, 4.82; N, 3.94, Found: C, 77.72; H, 4.84; N, 3.95; ESI/MS: m/z Calcd. For [M+Na] ^+^: 378.12, Found: 377.9.

**5-Chloro-3-{4-[2-oxo-2 phenylethoxy]benzylidene}indolin-2-one (3b)**

Yellow powder; 0.13 g, 31.81 % yield; mp: 189-191ºC; *R*_f_ = 0.32 (Pet.ether/ethyl acetate, 6:2.5); ^1^H NMR (400 MHz, DMSO-*d*_6_) δ 10.71 (s, 1H, N*H*), 8.05 (d, *J* = 7.3 Hz, 2H, Ar*H*), 7.91-7.84 (m, 1H, Ar*H*), 7.72-7.66 (m, 3H, Ar*H*), 7.64-7.53 (m, 3H, Ar*H*), 7.28 (dd, *J* = 8.3, 2.0 Hz, 1H, Ar*H*), 7.17 (d, *J* = 8.7 Hz, 2H, Ar*H*), 6.89 (d, *J* = 8.3 Hz, 1H, Ar*H*), 5.73 (s, 2H, CO-C*H_2_-*O); ^13^C NMR (100 MHz, DMSO-*d*_6_) δ 194.50, 167.56, 160.70, 141.86, 138.29, 135.06, 134.30, 131.94, 129.27, 128.32, 127.73, 126.97, 125.71, 123.50, 119.76, 115.50, 114.96, 110.96, 70.62; Anal. Calcd. for C_23_H_16_ClNO_3_ (389.08): C, 70.86; H, 4.14, N, 3.59, Found: C, 70.87; H, 4.15; N, 3.61; ESI/MS: m/z Calcd. For [M+Na] ^+^: 412.08, Found: 411.8.

**6-Chloro-3-{4-[2-oxo-2-phenylethoxy]benzylidene}indolin-2-one (3c)**

Dark yellow powder; 0.23 g, 55.5 % yield; mp: 199-201ºC; *R*_f_ = 0.30 (Pet.ether/ethyl acetate, 6:2.5); ^1^H NMR (400 MHz, DMSO-*d*_6_) δ 10.72 (s, 1H, N*H*), 8.05 (d, *J* = 8.5 Hz, 2H, Ar*H*), 7.70 (d, *J* = 8.5 Hz, 3H, Ar*H*), 7.65 (s, 1H, Ar*H*), 7.63-7.59 (m, 3H, Ar*H*), 7.16-7.09 (m, 2H, Ar*H*), 6.94-6.88 (m, 2H, Ar*H*), 5.72 (s, 2H, CO-C*H_2_-*O); ^13^C NMR (100 MHz, DMSO-*d*_6_) δ 194.20, 168.87, 159.49, 144.01, 141.36, 137.88, 136.86, 134.46, 133.92, 131.59, 127.92, 126.72, 124.54, 123.32, 120.81, 120.10, 115.08, 109.99, 70.27; Anal. Calcd. for C_23_H_16_ClNO_3_ (389.08): C, 70.86; H, 4.14, N, 3.59, Found: C, 70.88; H, 4.15; N, 3.62; ESI/MS: m/z Calcd. For [M-H]^-^: 388.08, Found: 388.09.

**(*E*/*Z*)-5-Fluoro-3-{4-[2-oxo-2-phenylethoxy]benzylidene}indolin-2-one (3d).**

***E* : *Z* ratio = 40 : 60.**

Yellow powder; 0.33 g, 67.55 % yield; mp: 223-226ºC; *R*_f_ = 0.28 (Petr.ether/ethyl acetate, 6:2.5);

*Z*-Diastereomer: ^1^H NMR (400 MHz, DMSO-*d*_6_) δ 10.59 (s, 1H, N*H*), 8.47 (d, *J* = 9 Hz, 1H, Ar*H*), 8.05 (t, *J* = 7.1 Hz, 1H, Ar*H*), 7.77 (s, 1H, Ar*H*), 7.71 (d, *J* = 7.3 Hz, 1H, Ar*H*), 7.63 – 7.60 (m, 2H, Ar*H*), 7.58 (d, *J* = 8.0 Hz, 1H, Ar*H*), 7.22-7.14 (m, 2H, Ar*H*), 7.04 (dd, *J* = 8.3, 2.1 Hz, 1H, Ar*H*), 6.93 (d, *J* = 8.7 Hz, 1H, Ar*H*), 6.88 (d, *J* = 3.5 Hz, 1H, Ar*H*), 6.86 (d, *J* = 3.4 Hz, 1H, Ar*H*), 5.77 (s, 2H, CO-C*H_2_-*O) ppm; ^13^C NMR (100 MHz, DMSO-*d*_6_) δ 191.31, 167.41, 159.56, 138.46, 137.63, 136.47, 134.56, 134.30, 133.88, 131.52, 127.91, 127.10, 126.57, 123.84, 115.85, 115.10, 114.53, 109.84, 70.20 ppm.

*E*-Diastereomer: ^1^H NMR (400 MHz, DMSO-*d*_6_) δ 10.55 (s, 1H, N*H*), 8.41 (d, *J* = 9 Hz, 1H, Ar*H*), 7.85 (t, *J* = 7.1 Hz, 1H, Ar*H*), 7.66 (s, 1H, Ar*H*), 7.41 (d, *J* = 7.3 Hz, 1H, Ar*H*), 7.40 – 7.36 (m, 2H, Ar*H*), 7.35 (d, *J* = 8.0 Hz, 1H, Ar*H*), 7.10-7.04 (m, 2H, Ar*H*), 6.99 (dd, *J* = 8.3, 2.1 Hz, 1H, Ar*H*), 6.80 (d, *J* = 8.7 Hz, 1H, Ar*H*), 6.78 (d, *J* = 3.5 Hz, 1H, Ar*H*), 6.76 (d, *J* = 3.4 Hz, 1H, Ar*H*), 5.73 (s, 2H, CO-C*H_2_-*O) ppm; ^13^C NMR (100 MHz, DMSO-*d*_6_) δ 194.19, 168.87, 160.22, 139.01, 137.63, 136.47, 134.56, 133.88, 131.69, 130.02, 128.86, 127.10, 126.59, 123.87, 116.08, 115.19, 114.62, 109.92, 70.27 ppm.

Anal. Calcd. for C_23_H_16_FNO_3_ (373.11): C, 73.99; H, 4.32; N, 3.75, Found: C, 74.00; H, 4.34; N, 3.76; ESI/MS: m/z Calcd. For [M+Na] ^+^: 396.11, Found: 395.8.

**5-Methoxy-3-{4-[2-oxo-2-phenylethoxy]benzylidene}indolin-2-one (3e)**

Yellow powder; 0.01 g, 26.41 % yield; mp: 196-198ºC; *R*_f_ = 0.34 (Pet.ether/ethyl acetate, 6:2.5); ^1^H NMR (400 MHz, DMSO-*d*_6_) δ 10.36 (s, 1H, N*H*), 8.05 (d, *J* = 7.3 Hz, 2H, Ar*H*), 7.69 (d, *J* = 8.8 Hz, 3H, Ar*H*), 7.61 (s, 1H, Ar*H*), 7.58 (d, *J* = 5.7 Hz, 2H, Ar*H*), 7.25-7.18 (m, 1H, Ar*H*), 7.14 (d, *J* = 8.8 Hz, 2H, Ar*H*), 6.83 (dd, *J* = 8.5, 2.2 Hz, 1H, Ar*H*), 6.78 (d, *J* = 8.5 Hz, 1H, Ar*H*), 5.71 (s, 2H, CO-C*H_2_-*O), 3.64 (s, 3H, OC*H_3_*); ^13^C NMR (100 MHz, DMSO-*d*_6_) δ 194.26, 168.94, 159.31, 154.03, 136.97, 136.20, 134.33, 133.92, 131.34, 128.90, 126.88, 126.33, 124.75, 121.95, 114.95, 114.45, 109.73, 108.72, 70.25, 55.34; Anal. Calcd. for C_24_H_19_NO_4_ (385.13): C, 74.79; H, 4.97; N, 3.63, Found: C, 74.80; H, 4.99; N, 3.64; ESI/MS: m/z Calcd. For [M+Na] ^+^: 408.13, Found: 407.8.

**3-{4-[2-(4-Bromophenyl)-2-oxoethoxy]benzylidene}indolin-2-one (3f)**

Yellow powder; 0.13 g, 30.22 % yield; mp: 265-267ºC; *R*_f_ = 0.32 (Pet.ether/ethyl acetate, 6:2.5);^1^H NMR (400 MHz, DMSO-*d*_6_) δ 10.55 (s, 1H, N*H*), 8.13-7.88 (m, 2H, Ar*H*), 7.87-7.68 (m, 4H, Ar*H*), 7.67-7.60 (m, 1H, Ar*H*), 7.58 (s, 1H, Ar*H*), 7.31-7.02 (m, 3H, Ar*H*), 7- 6.68 (m, 2H, Ar*H*), 5.69 (s, 2H, CO-C*H_2_-*O); ^13^C NMR (100 MHz, DMSO-*d*_6_) δ 193.59, 168.90, 159.17, 142.70, 140.29, 135.89, 133.30, 131.93, 129.94, 127.98, 127.05, 125.76, 124.27, 122.09, 121.04, 119.30, 114.98, 110.07, 70.23; Anal. Calcd. for C_23_H_16_BrNO_3_ (433.03): C, 63.61; H, 3.71; N, 3.23, Found: C, 63.62; H, 3.72; N, 3.25; ESI/MS: m/z Calcd. For [M-H]^-^: 432.03, Found: 431.4.

**6-Chloro-3-{4-[2-(4-bromophenyl)-2-oxoethoxy]benzylidene}indolin-2-one (3g)**

Dark orange powder; 0.16 g, 35.31 % yield; mp: 249-251ºC; *R*_f_ = 0.31 (Pet.ether/ethyl acetate, 6:2.5); ^1^H NMR (400 MHz, DMSO-*d*_6_) δ 10.73 (s, 1H, N*H*), 8.44 (d, *J* = 8.8 Hz, 2H, Ar*H*), 7.97 (d, *J* = 8.4 Hz, 2H, Ar*H*), 7.85-7.77 (m, 3H, Ar*H*), 7.70 (d, *J* = 8.1 Hz, 1H, Ar*H*), 7.09 (d, *J* = 8.8 Hz, 2H, Ar*H*), 7.05-7.00 (m, 1H, Ar*H*), 6.87-6.80 (m, 1H, Ar*H*), 5.69 (s, 2H, CO-C*H_2_-*O);); ^13^C NMR (100 MHz, DMSO-*d*_6_) δ 193.98, 169.30, 159.80, 144.47, 137.27, 134.91, 134.06, 132.38, 132.03, 130.40, 128.44, 127.24, 125.03, 123.75, 121.25, 120.54, 115.54, 110.45, 70.70; Anal. Calcd. for C_23_H_15_BrClNO_3_ (466.99): C, 58.94; H, 3.23; N, 2.99, Found: C, 58.96; H, 3.24; N, 3.00; ESI/MS: m/z Calcd. For [M-H]^-^: 465.99, Found: 465.6.

**3-{4-[2-(4-Methoxyphenyl)-2-oxoethoxy]benzylidene}indolin-2-one (3h)**

Yellow powder; 0.22 g, 59.37 % yield; mp: 192-194ºC; ; *R*_f_ = 0.37 (Pet.ether/ethyl acetate, 6:2.5); ^1^H NMR (400 MHz, DMSO-*d*_6_) δ 10.74 (s, 1H, N*H*), 8.05 (d, *J* = 8.6 Hz, 2H, Ar*H*), 7.72 (d, *J* = 8.6 Hz, 2H, Ar*H*), 7.67 (s, 1H, Ar*H*), 7.65 (d, *J* = 3.4 Hz, 1H, Ar*H*), 7.13 (d, *J* = 8.8 Hz, 4H, Ar*H*), 7.10-7.03 (m, 1H, Ar*H*), 6.96 (dd, *J* = 8.3, 2.0 Hz, 1H, Ar*H*), 6.91 (d, *J* = 2.0 Hz, 1H, Ar*H*), 5.66 (s, 2H, CO-C*H_2_-*O), 3.90 (s, 3H, OC*H_3_*); ^13^C NMR (100 MHz, DMSO-*d*_6_) δ 192.41, 168.86, 163.66, 159.57, 143.99, 136.86, 134.45, 131.58, 130.29, 127.18, 124.32, 123.31, 120.80, 120.09, 119.82, 115.05, 114.10, 109.98, 69.97, 55.65; Anal. Calcd. for C_24_H_19_NO_4_ (385.13): C, 74.79; H, 4.97; N, 3.63, Found: C, 74.80; H, 4.99; N, 3.64; ESI/MS: m/z Calcd. For [M+Cl] ^-^: 420.03, Found: 419.2.

**5-Chloro-3-{4-[2-(4-methoxyphenyl)-2-oxoethoxy]benzylidene}indolin-2-one (3i)**

Yellow powder; 0.22 g, 51.31 % yield; mp: 183-185ºC; *R*_f_ = 0.29 (Pet.ether/ethyl acetate, 6:2.5); ^1^H NMR (400 MHz, DMSO-*d*_6_) δ 10.55 (s, 1H, N*H*), 8.03 (d, *J* = 8.9 Hz, 2H, Ar*H*), 7.74-7.63 (m, 2H, Ar*H*), 7.58 (s, 1H, Ar*H*), 7.22 (t, *J* = 7.8 Hz, 1H, Ar*H*), 7.11 (d, *J* = 8.9 Hz, 4H, Ar*H*), 6.92-6.83 (m, 2H, Ar*H*), 5.63 (s, 2H, CO-C*H_2_-*O), 3.87 (s, 3H, OC*H_3_*); ^13^C NMR (100 MHz, DMSO-*d*_6_) δ 192.96, 169.34, 164.09, 159.87, 143.11, 140.70, 136.36, 134.67, 130.72, 130.17, 127.63, 126.11, 124.61, 122.52, 121.57, 115.38, 114.53, 110.48, 70.39 , 56.08; Anal. Calcd. for C_24_H_18_ClNO_4_ (419.09): C, 68.66; H, 4.32; N, 3.34, Found: C, 68.67; H, 4.33; N, 3.36; ESI/MS: m/z Calcd. For [M-H] ^-^: 418.08, Found: 417.5.

**(*E*/*Z*)-6-Chloro-3-{4-[2-(4-methoxyphenyl)-2-oxoethoxy]benzylidene}indolin-2-one (3j). *E* : *Z* ratio = 38.5 : 61.5.**

Yellow powder; 0.22 g, 51.31 % yield; mp: 165-167ºC; *R*_f_ = 0.27 (Pet.ether/ethyl acetate, 6:2.5);

*Z*-Diastereomer: ^1^H NMR (400 MHz, DMSO-*d*_6_) δ 10.73 (s, 1H, N*H*), 8.49 (d, *J* = 9 Hz, 1H, Ar*H*), 8.06 (d, *J* = 2.9 Hz, 1H, Ar*H*), 8.04 (d, *J* = 2.9 Hz, 1H, Ar*H*), 7.92 (s, 1H, Ar*H*), 7.83 (dd, *J* = 9.7, 2.2 Hz, 1H, Ar*H*), 7.72 (d, *J* = 8.7 Hz, 1H, Ar*H*), 7.37-7.15 (m, 4H, Ar*H*), 6.91 (d, *J* = 8.4 Hz, 1H, Ar*H*), 6.84 (d, *J* = 8.4 Hz, 1H, Ar*H*), 5.68 (s, 2H, CO-C*H_2_-*O), 3.90 (s, 3H, OC*H_3_*) ppm; ^13^C NMR (100 MHz, DMSO-*d*_6_) δ 192.36, 167.13, 161.61, 159.70, 140.97, 138.74, 137.87, 133.30, 130.27, 129.19, 127.30, 127.19, 124.87, 122.80, 121.45, 114.51, 114.08, 110.52, 69.91, 55.60 ppm.

*E*-Diastereomer: ^1^H NMR (400 MHz, DMSO-*d*_6_) δ 10.69 (s, 1H, N*H*), 8.44 (d, *J* = 9 Hz, 1H, Ar*H*), 8.00 (d, *J* = 2.9 Hz, 1H, Ar*H*), 7.97 (d, *J* = 2.9 Hz, 1H, Ar*H*), 7.69 (s, 1H, Ar*H*), 7.63 (dd, *J* = 9.7, 2.2 Hz, 1H, Ar*H*), 7.59 (d, *J* = 8.7 Hz, 1H, Ar*H*), 7.14-7.07 (m, 4H, Ar*H*), 6.76 (d, *J* = 8.4 Hz, 1H, Ar*H*), 6.70 (d, *J* = 8.4 Hz, 1H, Ar*H*), 5.66 (s, 2H, CO-C*H_2_-*O), 3.86 (s, 3H, OC*H_3_*) ppm; ^13^C NMR (100 MHz, DMSO-*d*_6_) δ 192.43, 168.56, 163.63, 160.37, 141.42, 138.86, 138.02, 134.62, 131.51, 129.61, 127.6, 127.19, 125.27, 123.02, 121.65, 115.05, 114.08, 111.38, 69.97, 55.63 ppm.

Anal. Calcd. for C_24_H_18_ClNO_4_ (419.09): C, 68.66; H, 4.32; N, 3.34, Found: C, 68.69; H, 4.33; N, 3.35; ESI/MS: m/z Calcd. For [M+Na] ^+^: 442.09, Found: 441.8.


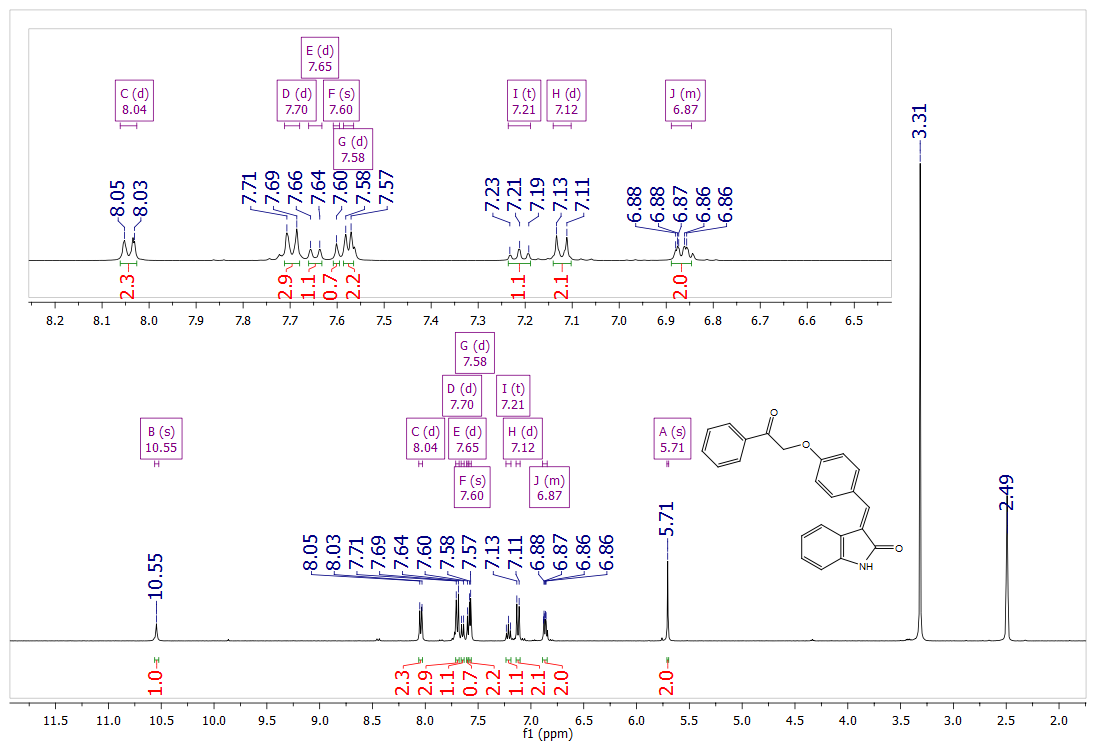


Figure S1. ^1^H NMR spectrum of compound **3a**.


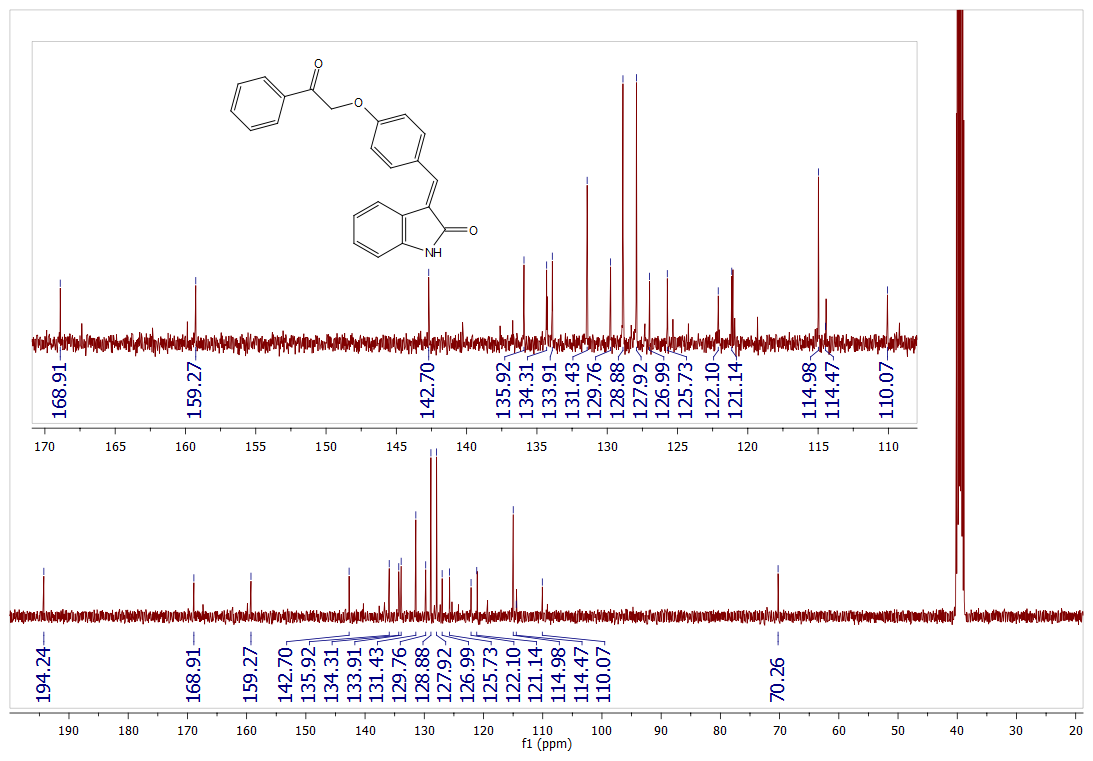


Figure S2. ^13^C NMR spectrum of compound **3a**.


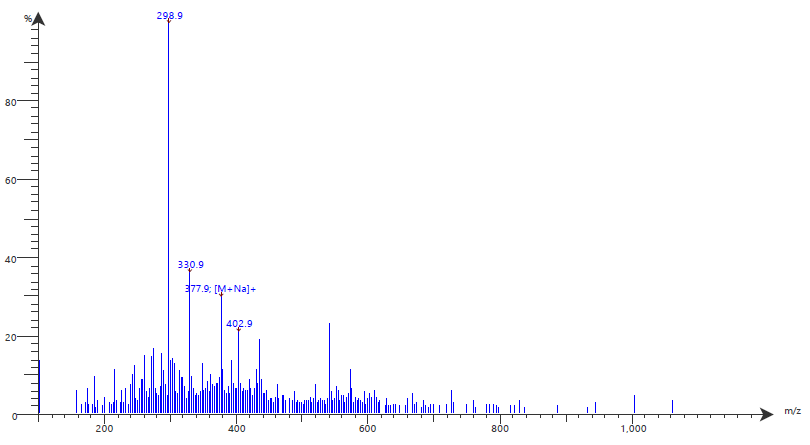


Figure S3. MS (ESI^+^) spectrum of compound **3a**.


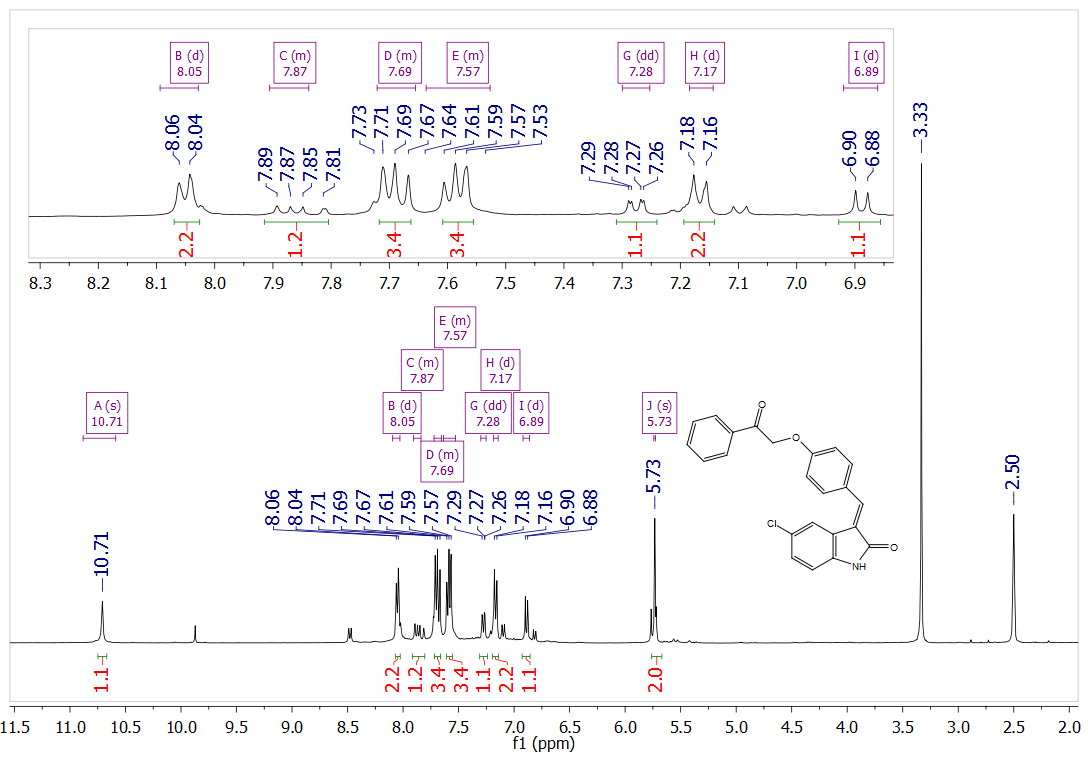


Figure S4. ^1^H NMR spectrum of compound **3b**.


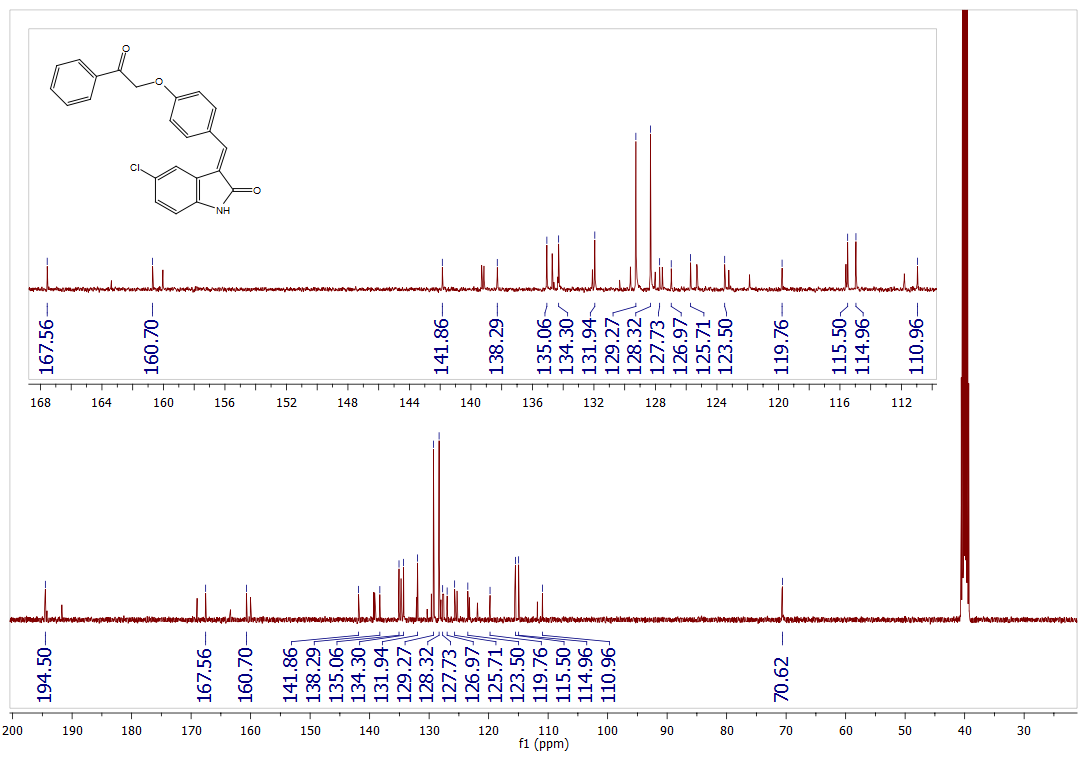


Figure S5. ^13^C NMR spectrum of compound **3b**.


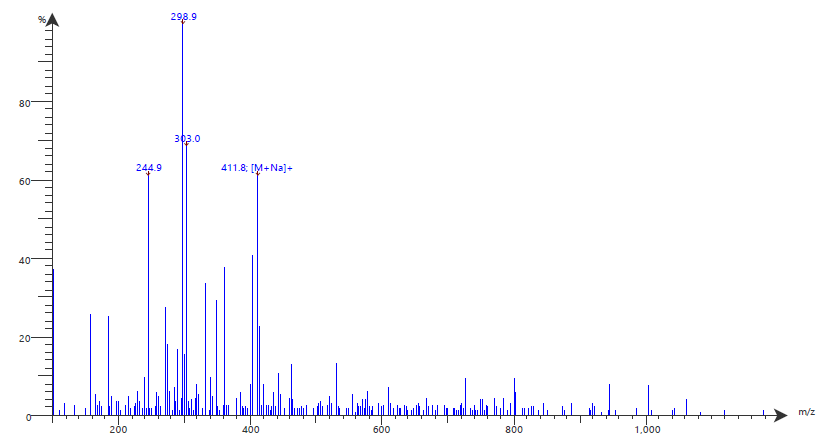

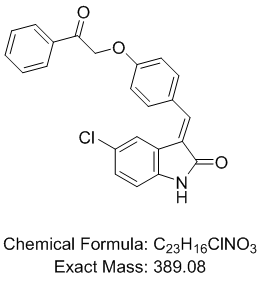


Figure S6. MS (ESI^+^) spectrum of compound **3b**.


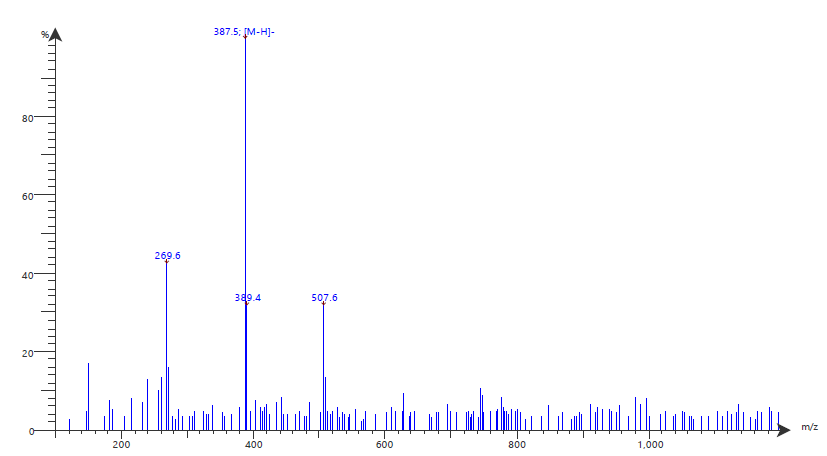

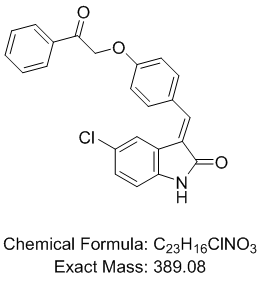


Figure S7. MS (ESI^-^) spectrum of compound **3b**.


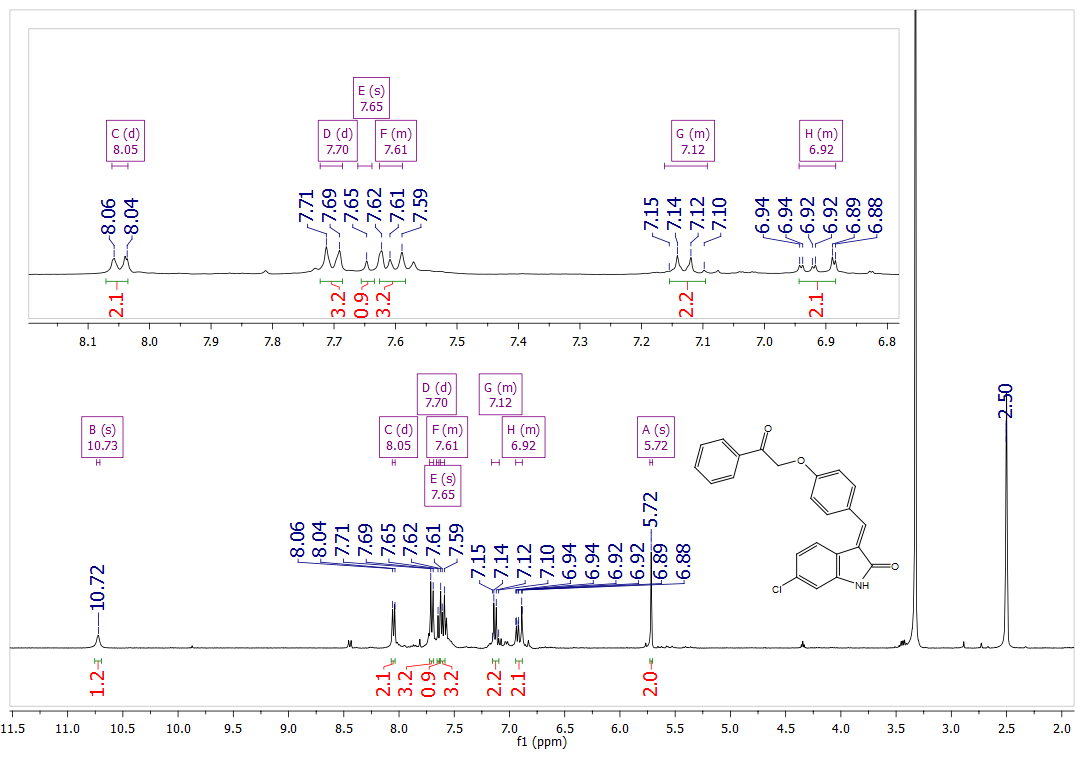


Figure S8. ^1^H NMR spectrum of compound **3c**.


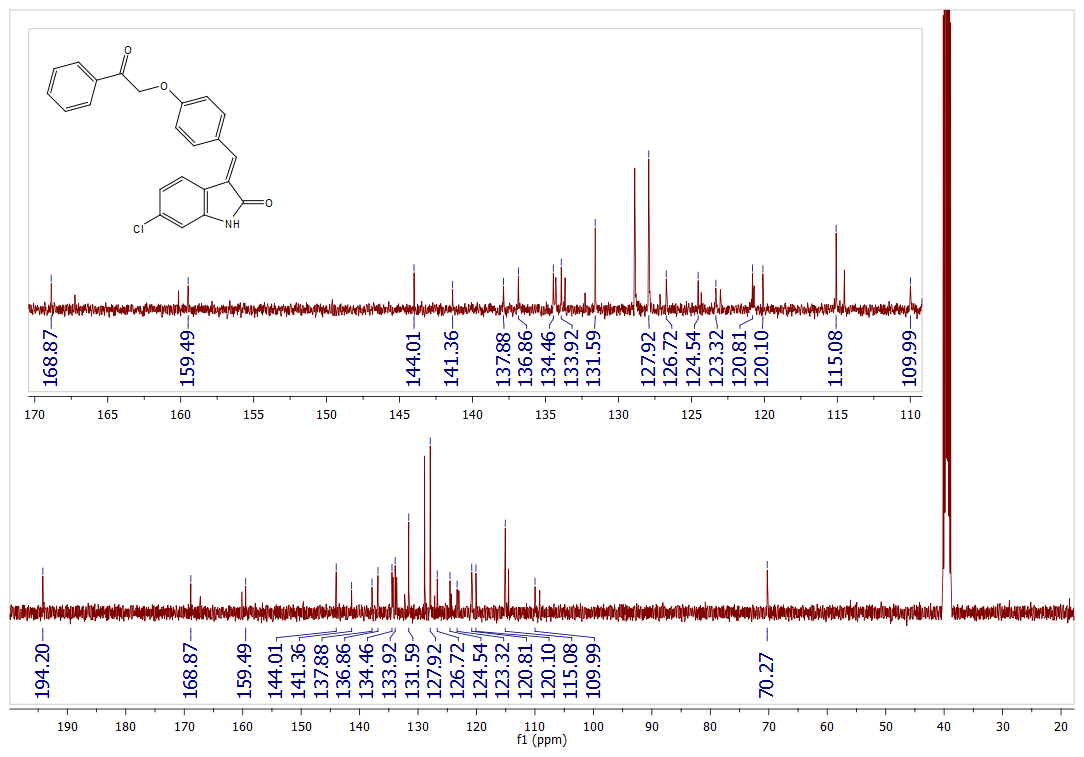


Figure S9. ^13^C NMR spectrum of compound **3c**.

Figure S10. MS (ESI^-^) spectrum of compound **3c**.


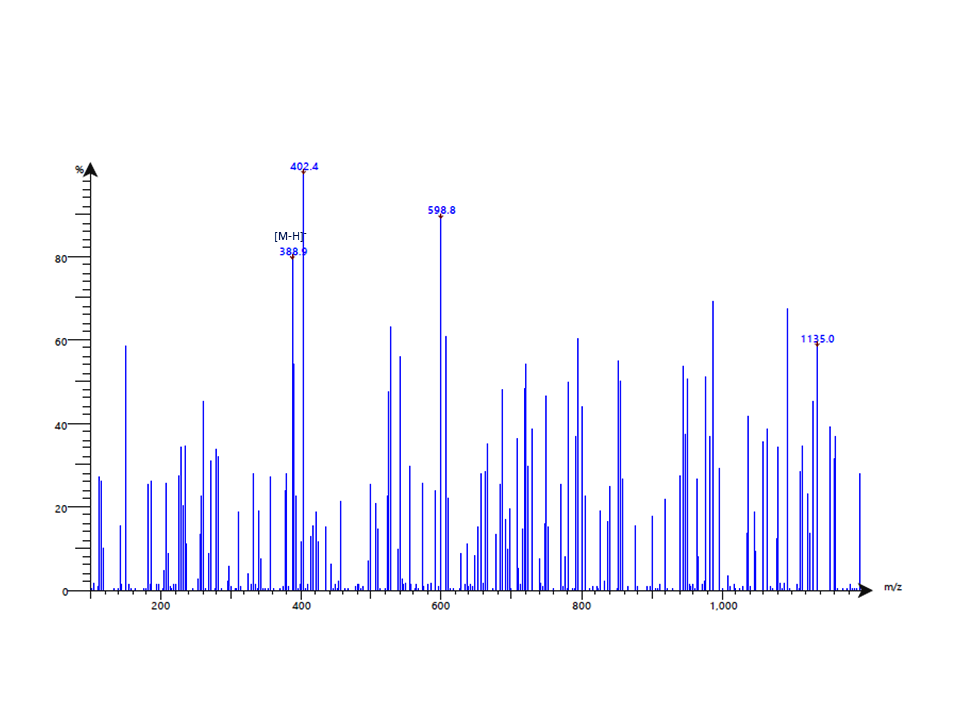


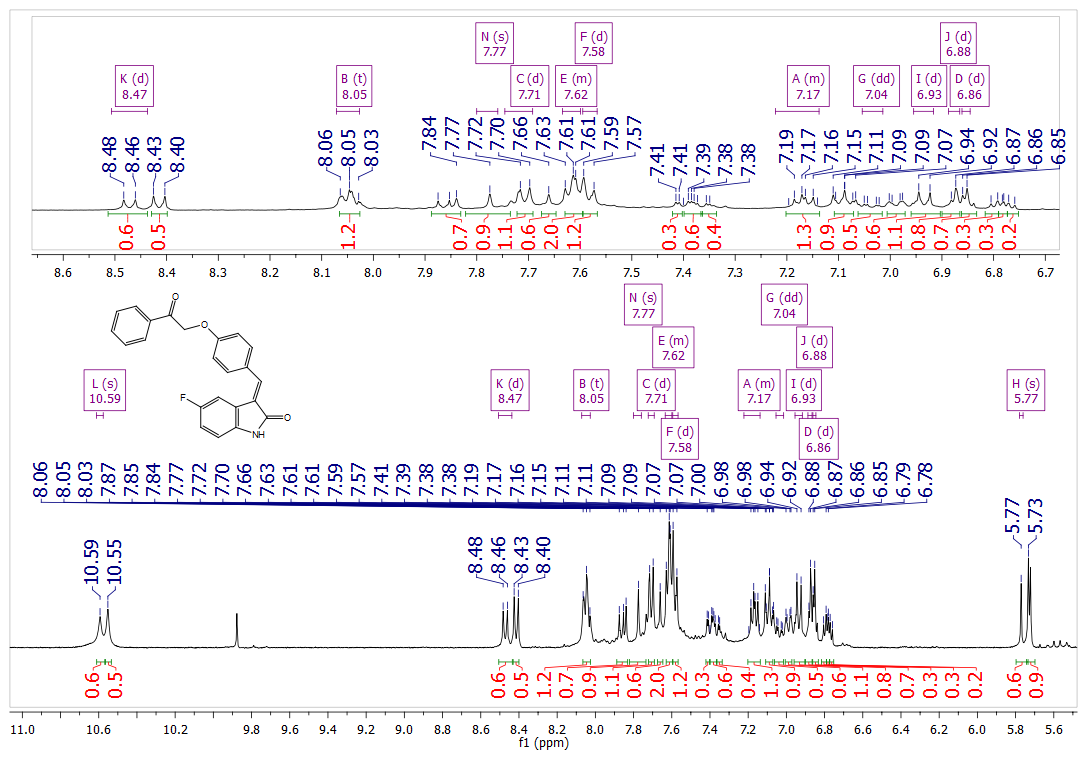


Figure S11. ^1^H NMR spectrum of compound **3d (*E/Z*)**.


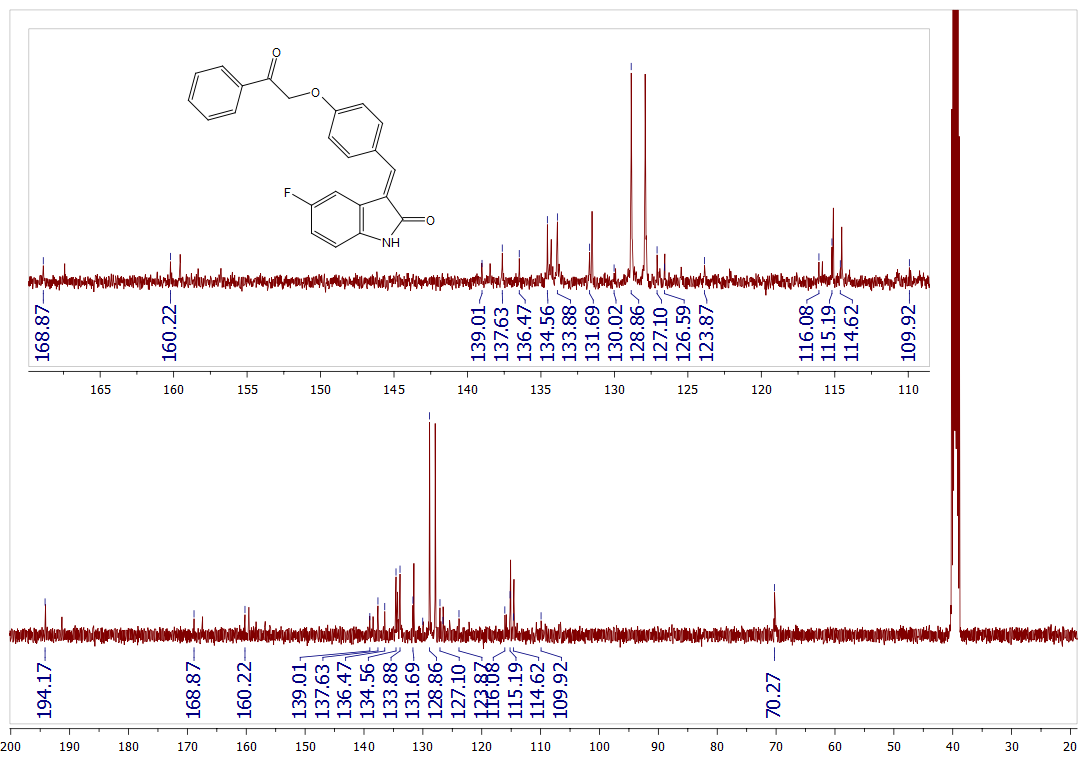


Figure S12. ^13^C NMR spectrum of compound **3d (*E* isomer)**.


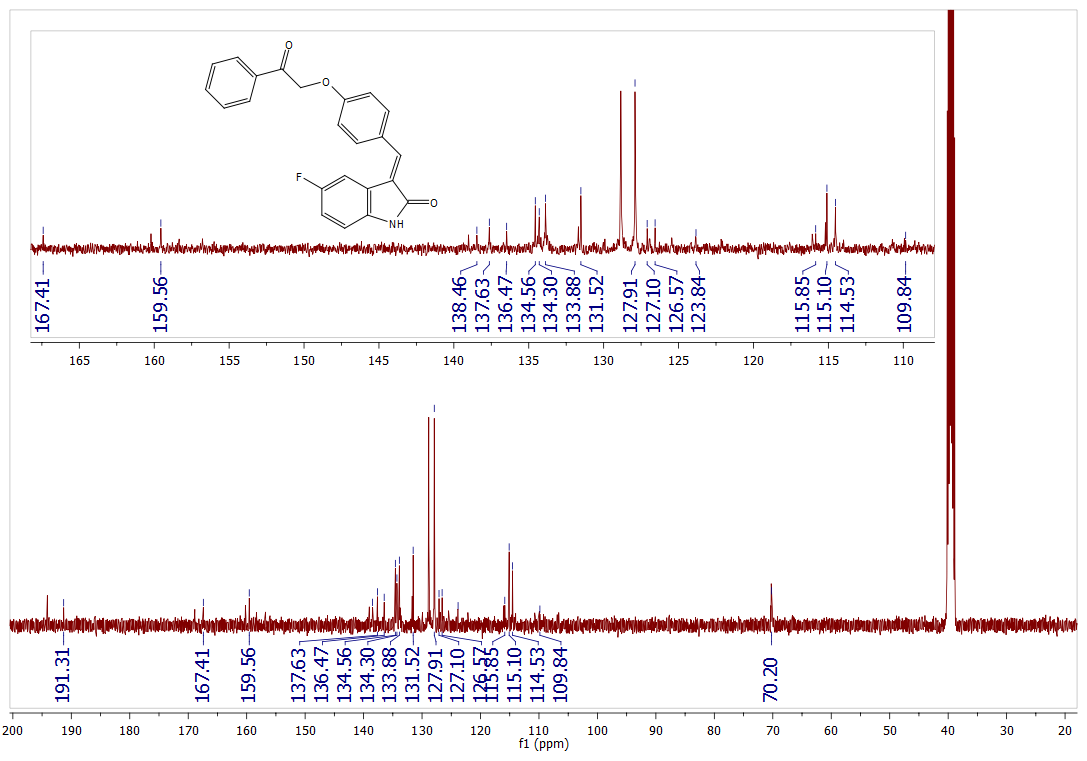


Figure S13. ^13^C NMR spectrum of compound **3d (*Z* isomer)**.

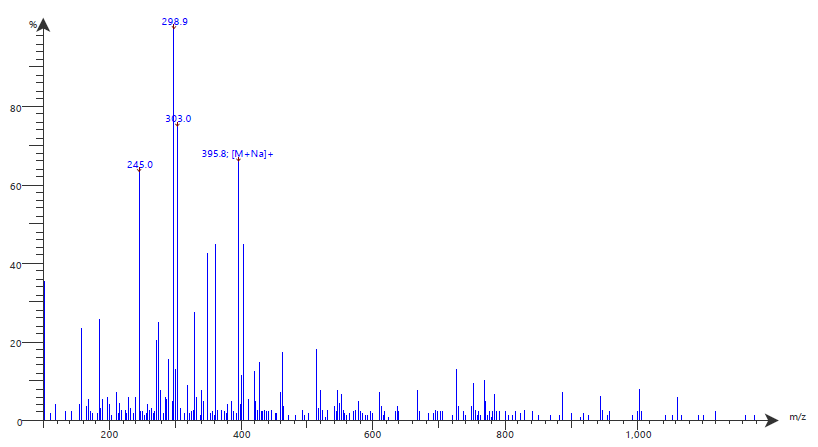

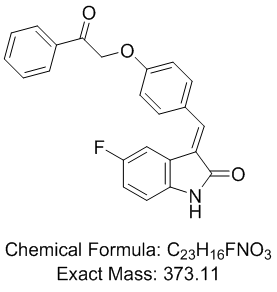


Figure S14. MS (ESI^+^) spectrum of compound **3d**.


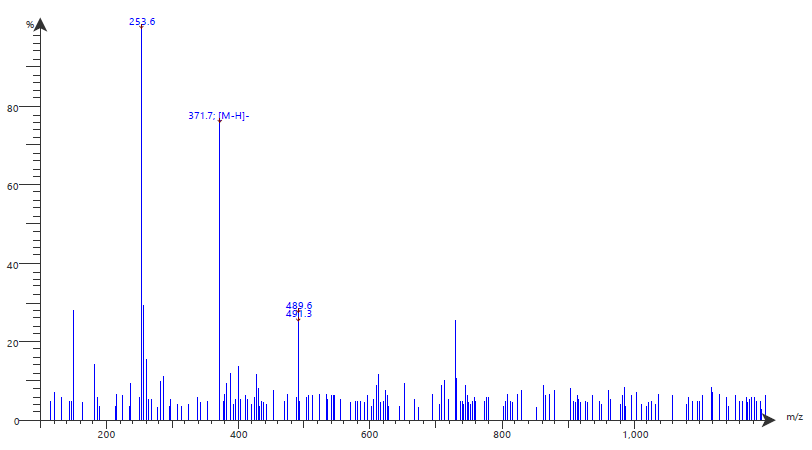


Figure S15. MS (ESI^-^) spectrum of compound **3d**.


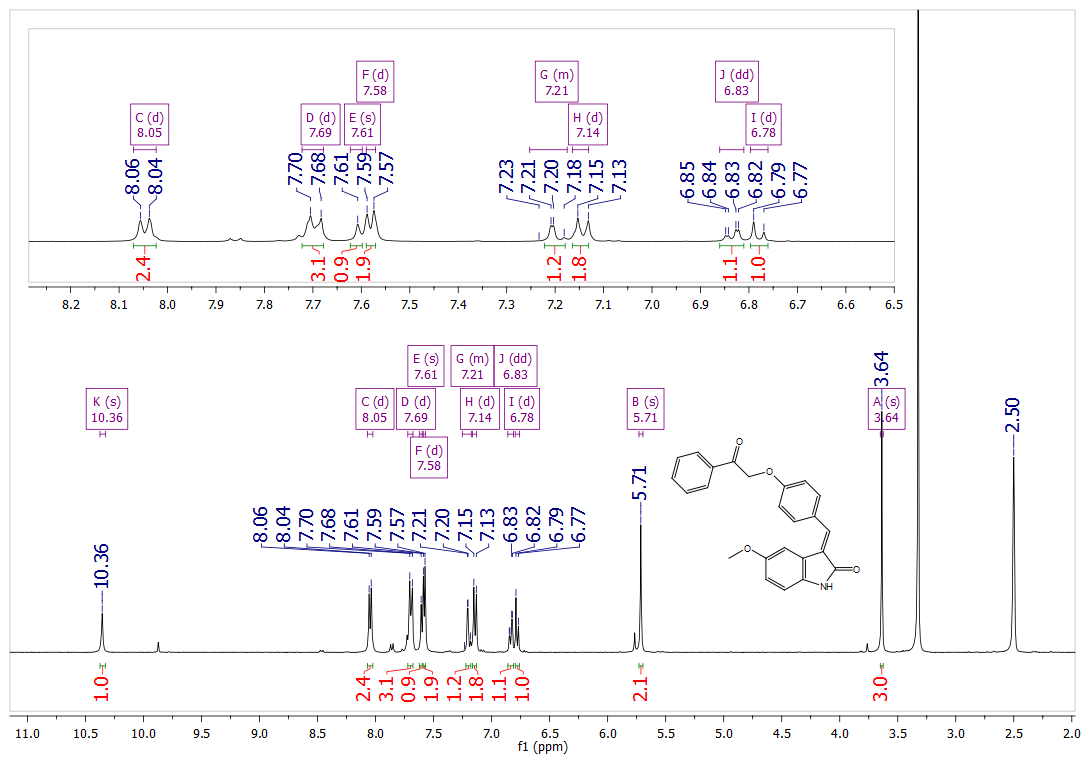


Figure S16. ^1^H NMR spectrum of compound **3e**.


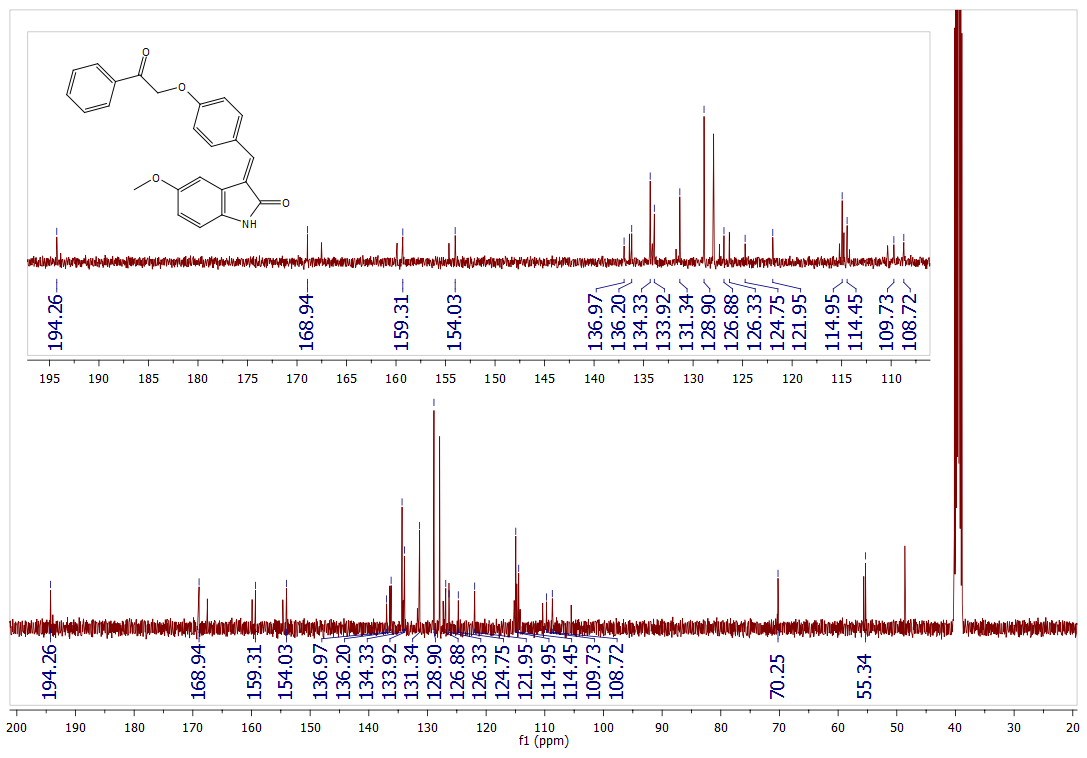


Figure S17. ^13^C NMR spectrum of compound **3e**.


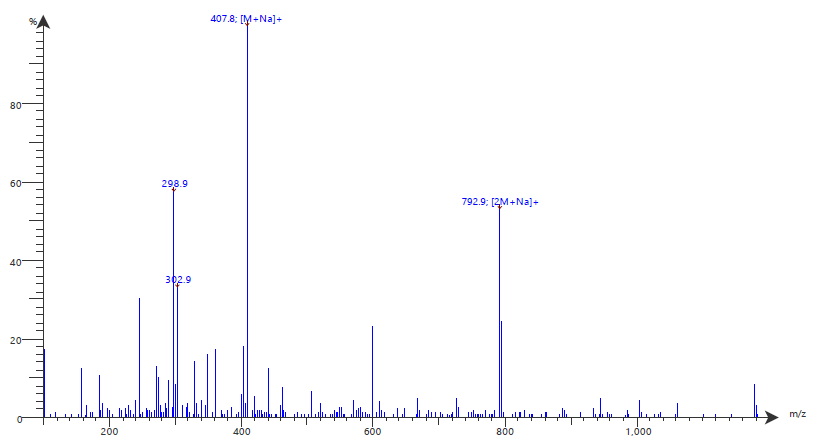

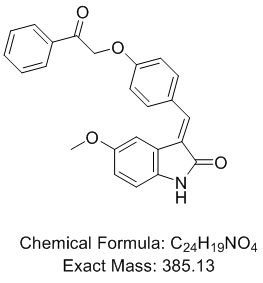


Figure S18. MS (ESI^+^) spectrum of compound **3e**.


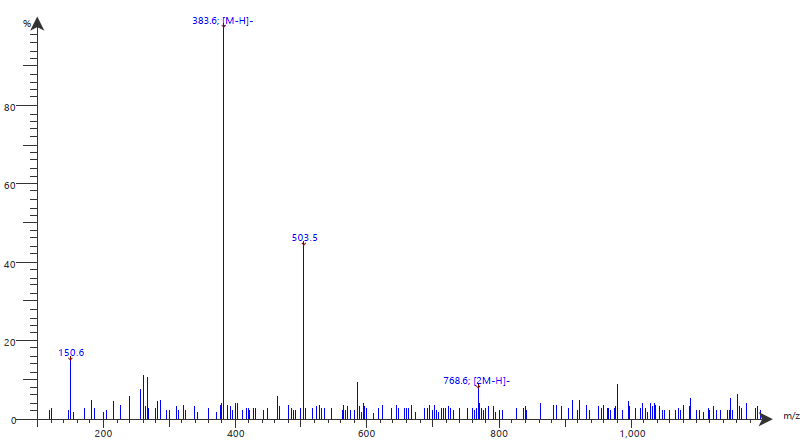


Figure S19. MS (ESI^-^) spectrum of compound **3e**.


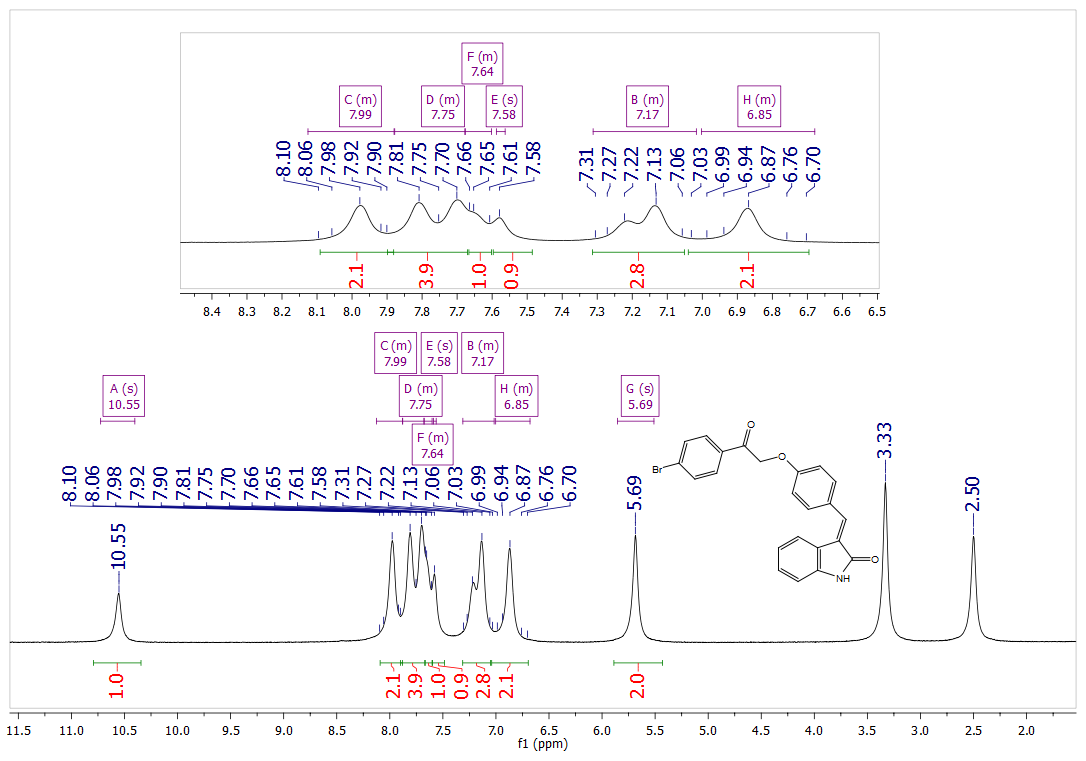


Figure S20. ^1^H NMR spectrum of compound **3f**.


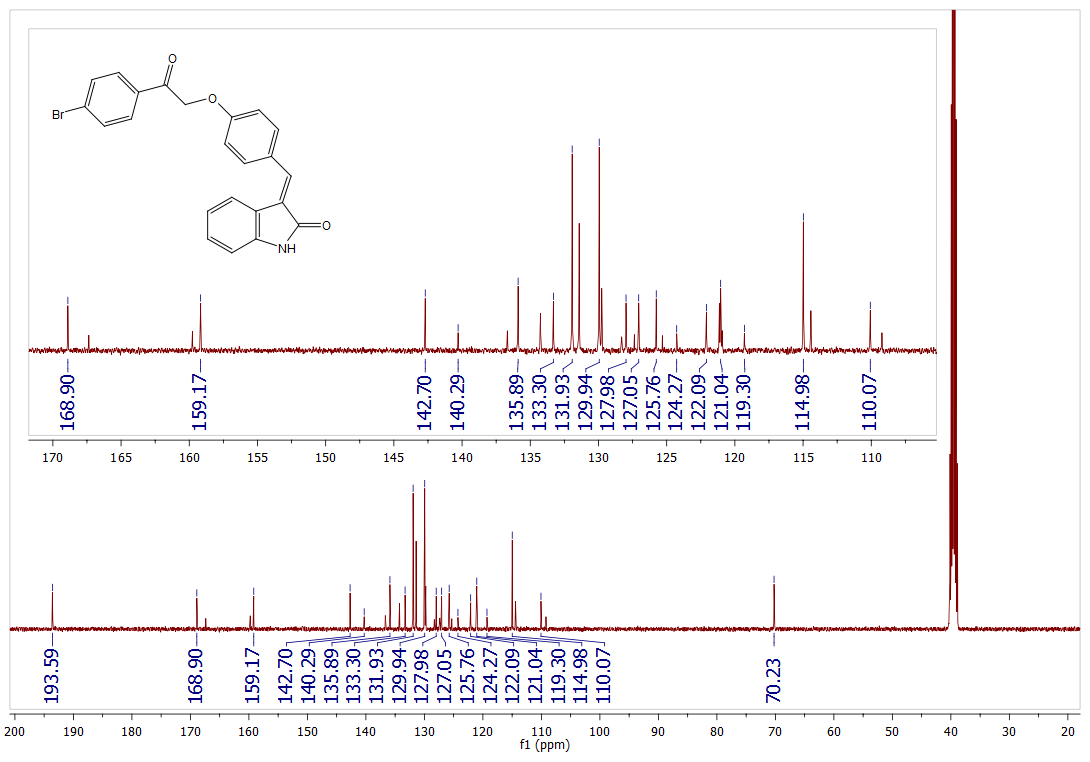


Figure S21. ^13^C NMR spectrum of compound **3f**.


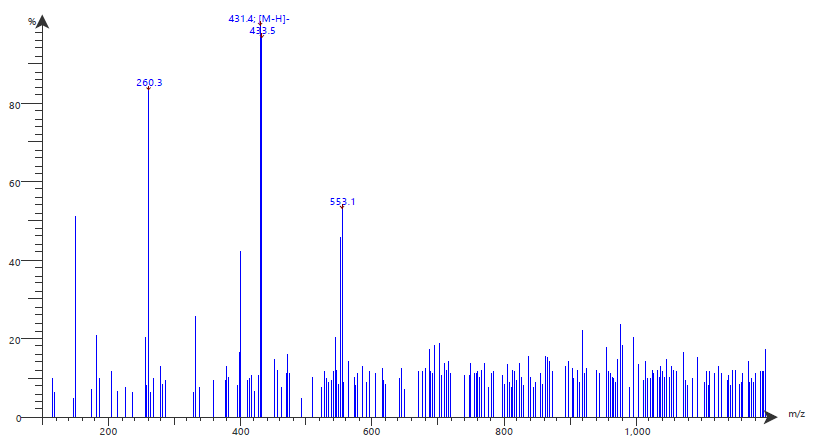


Figure S22. MS (ESI^-^) spectrum of compound **3f**.


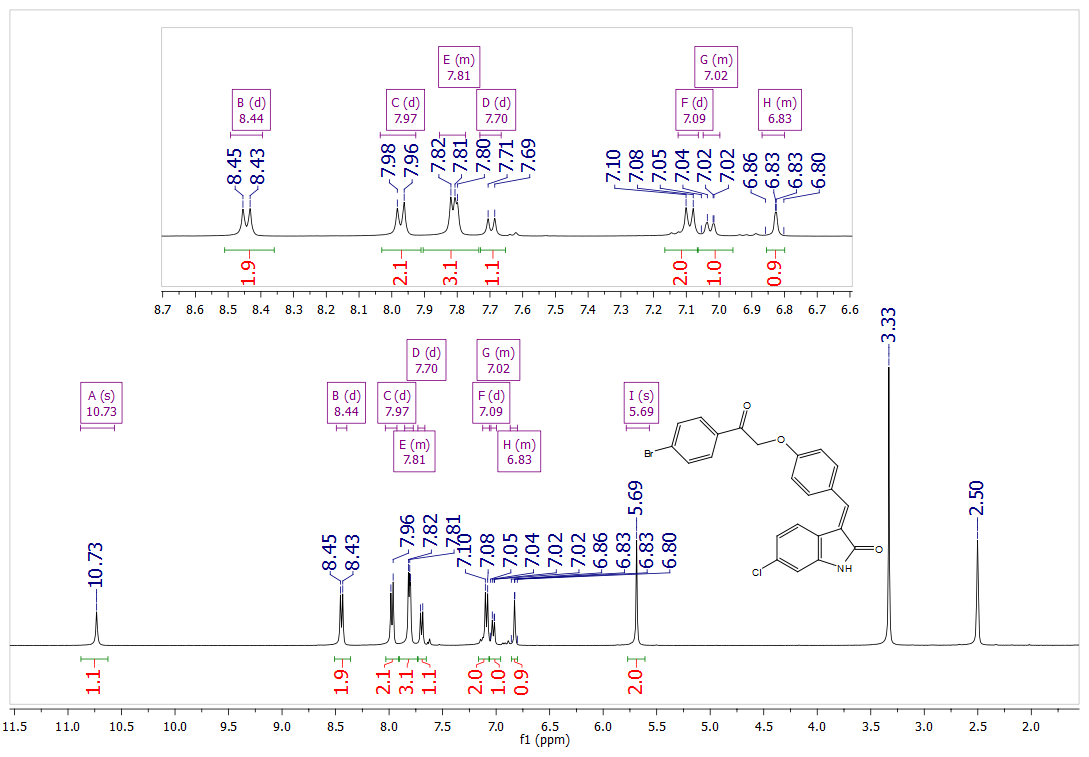


Figure S23. ^1^H NMR spectrum of compound **3g**.


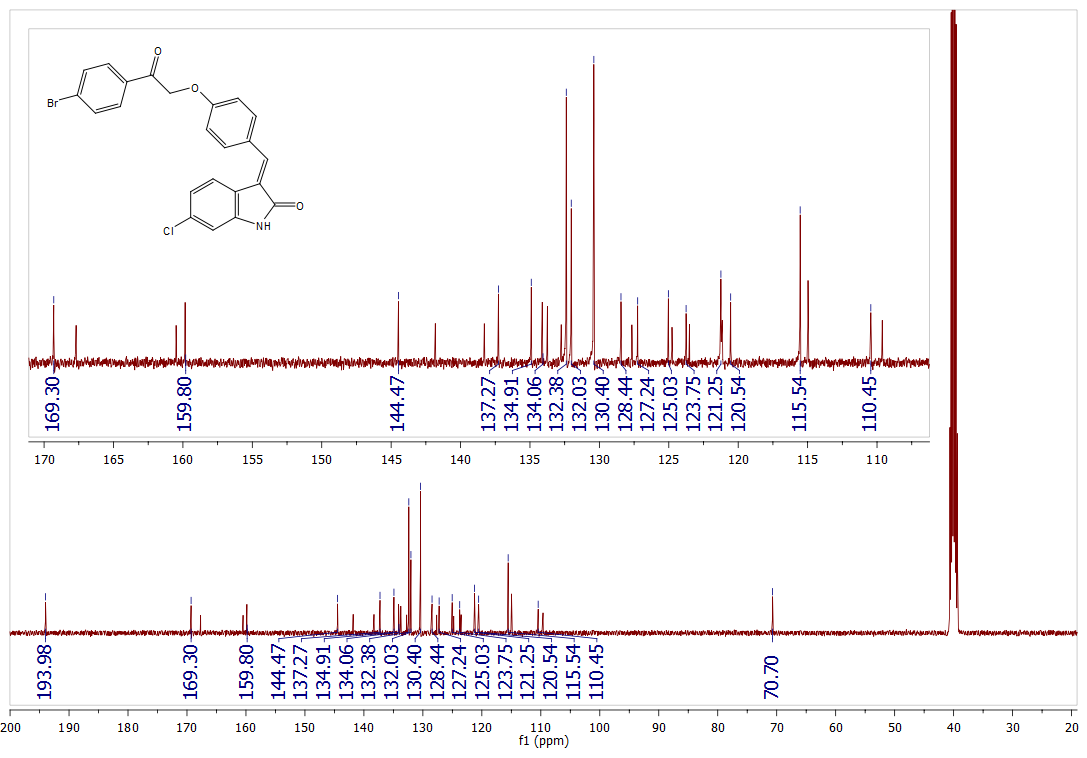


Figure S24. ^13^C NMR spectrum of compound **3g**.


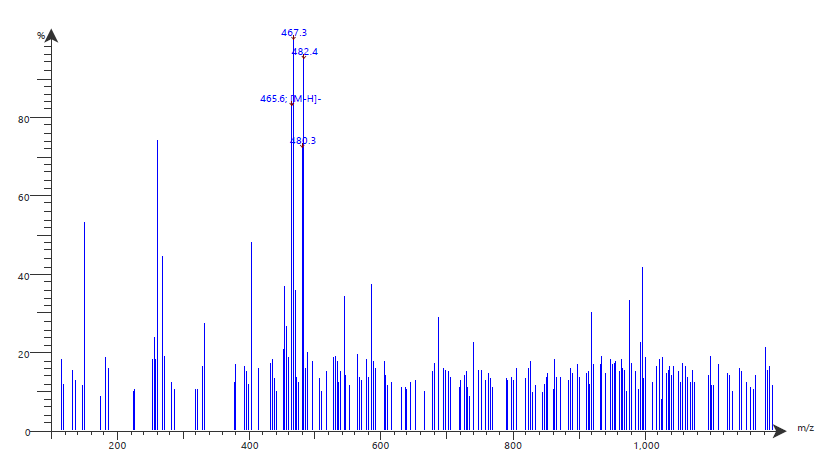


Figure S25. MS (ESI^-^) spectrum of compound **3g**.


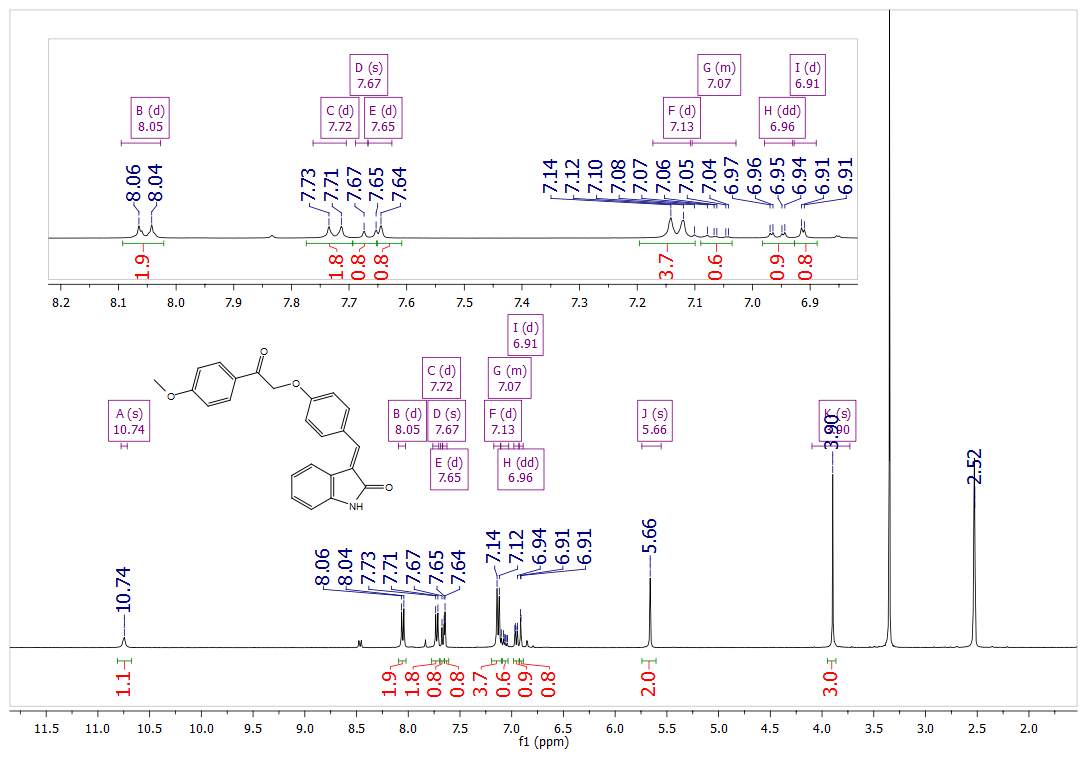


Figure S26. ^1^H NMR spectrum of compound **3h**.


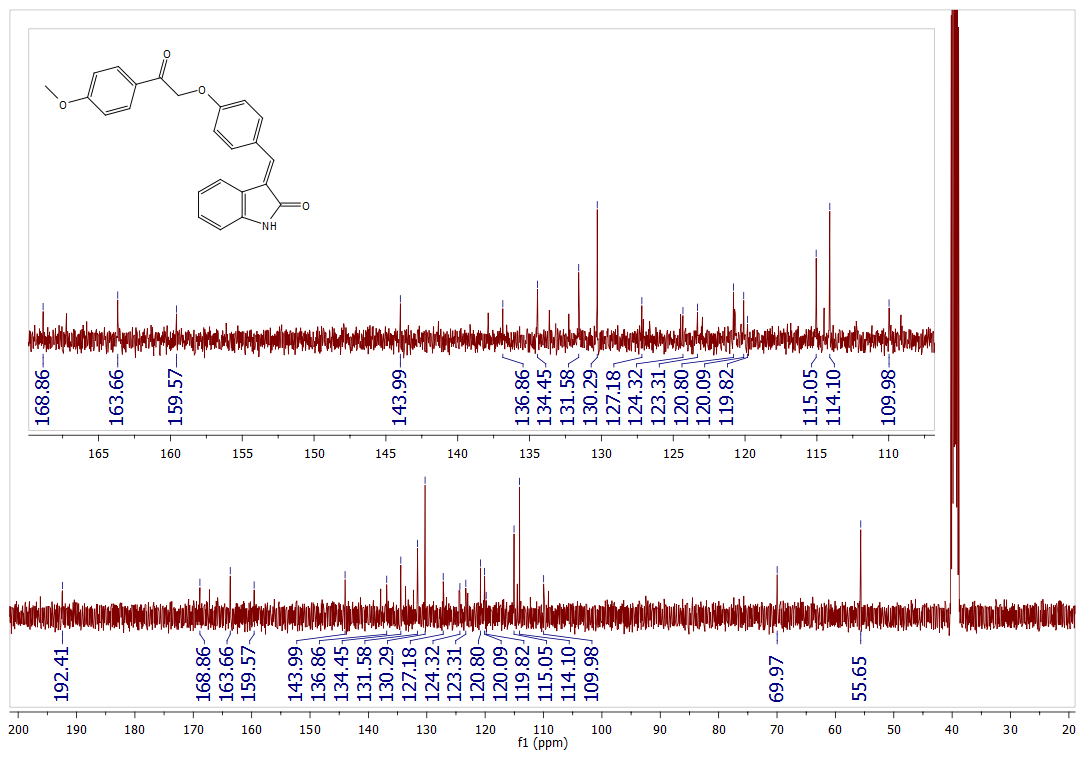


Figure S27. ^13^C NMR spectrum of compound **3h**.


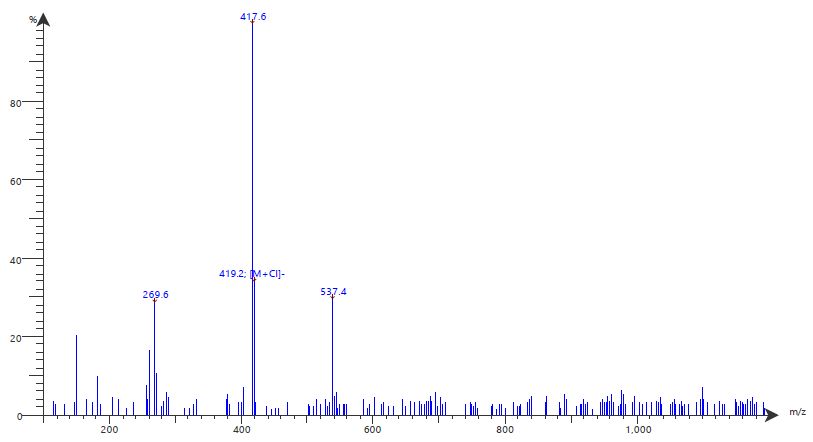


Figure S28. MS (ESI^-^) spectrum of compound **3h**.


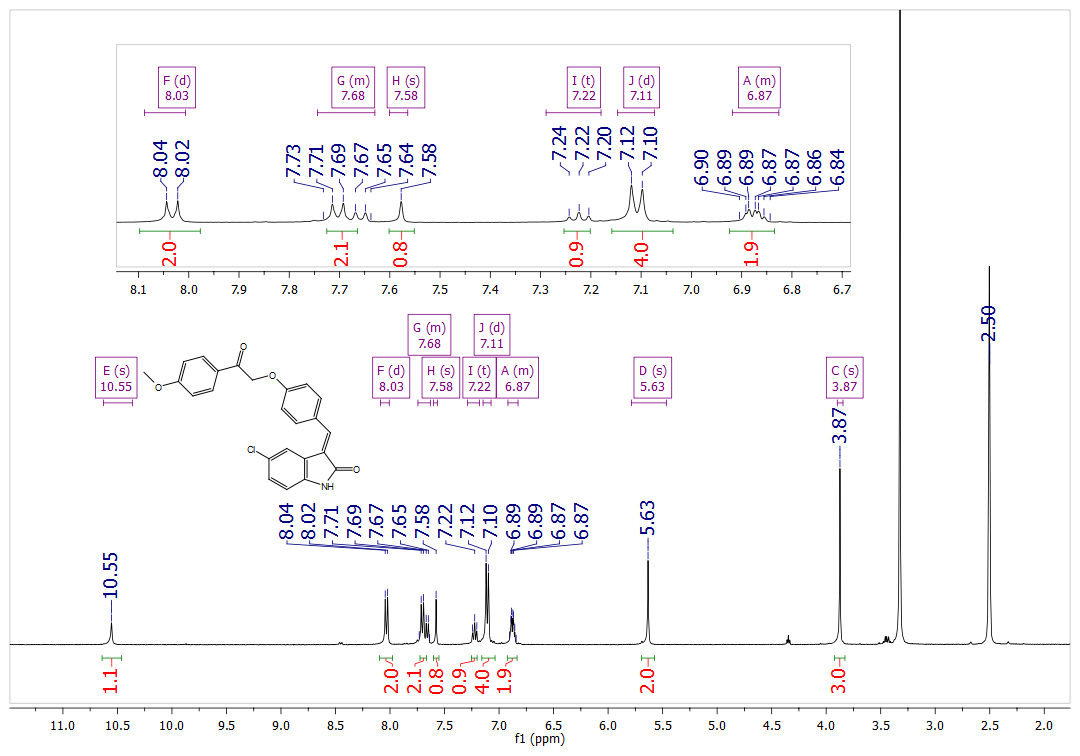


Figure S29. ^1^H NMR spectrum of compound **3i**.


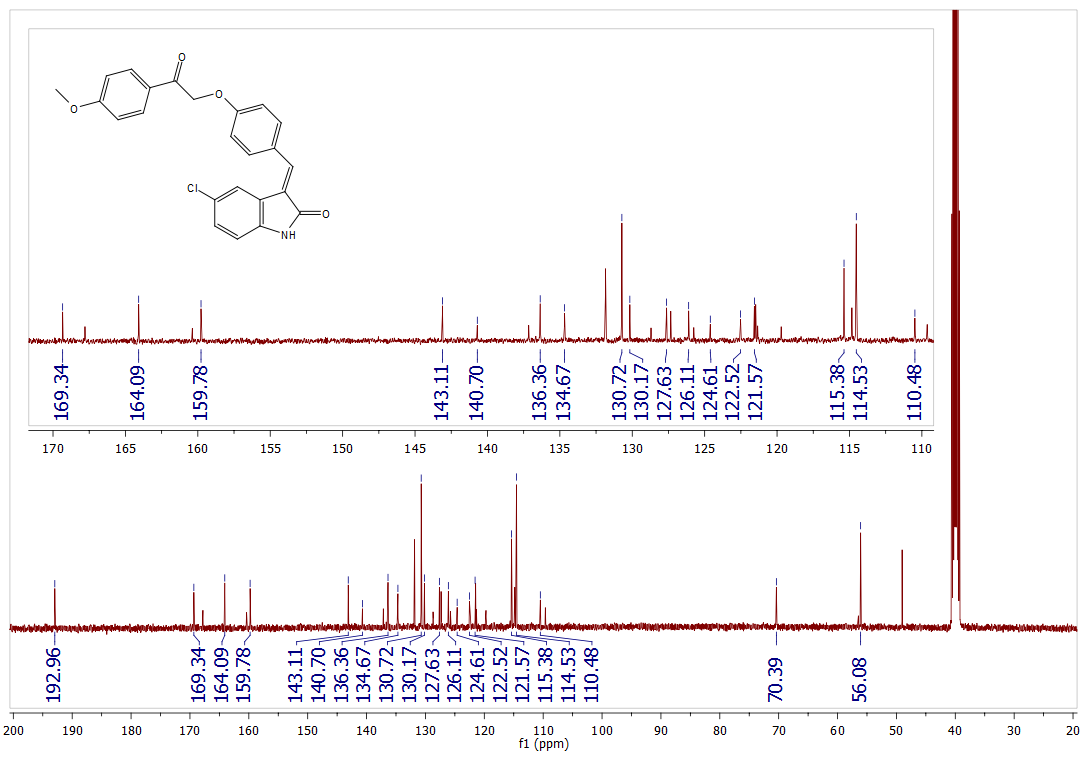


Figure S30. ^13^C NMR spectrum of compound **3i**.

Figure S31. MS (ESI^-^) spectrum of compound **3i**.


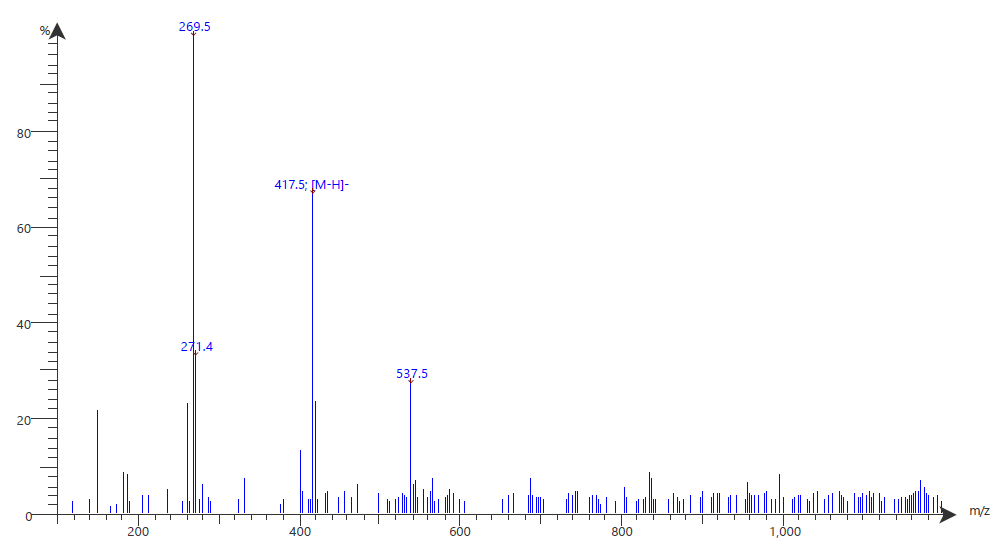


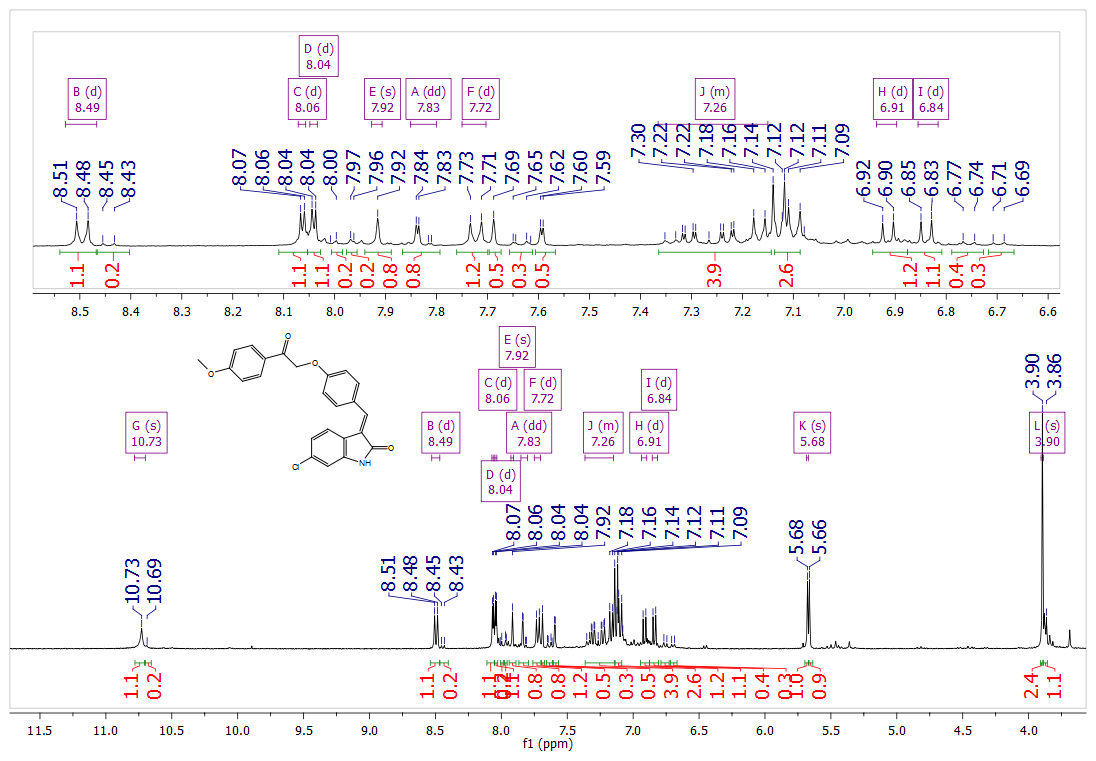


Figure S32. ^1^H NMR spectrum of compound **3j** (**E/Z**).


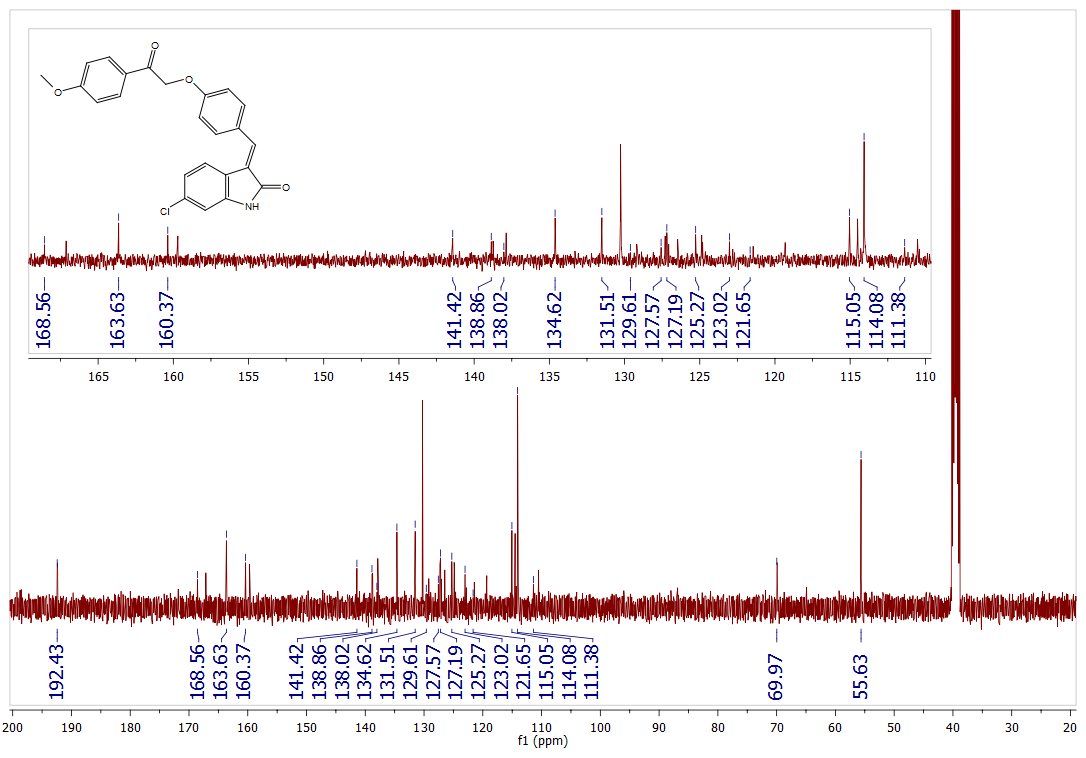


Figure S33. ^13^C NMR spectrum of compound **3j (*E* isomer)**.


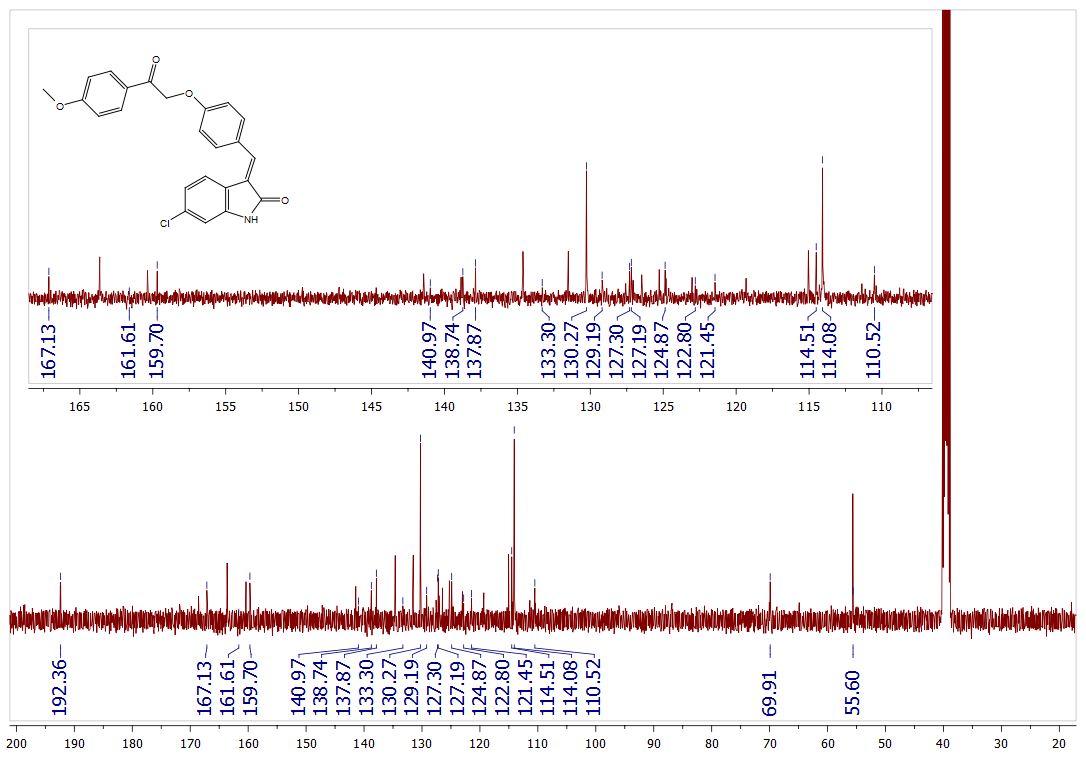


Figure S34. ^13^C NMR spectrum of compound **3j (*Z* isomer)**.


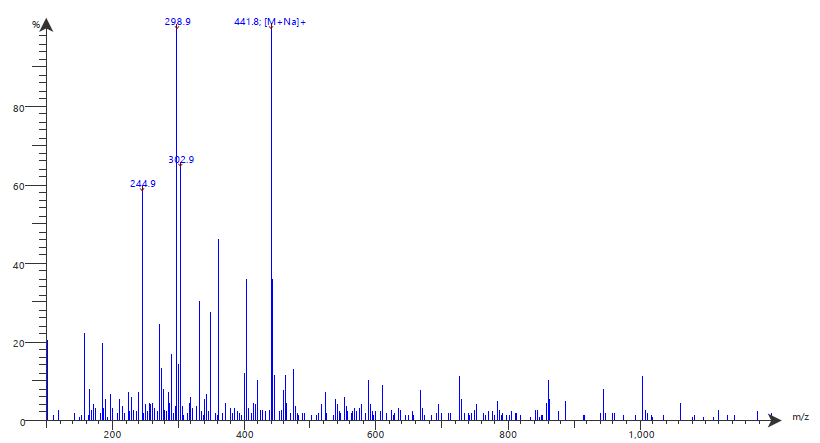


Figure S35. MS (ESI^+^) spectrum of compound **3j**.


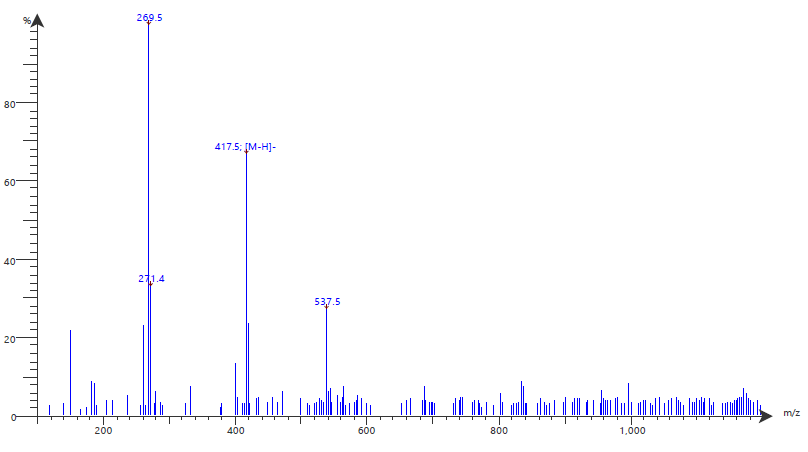

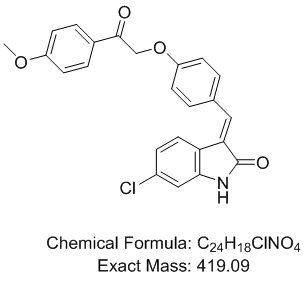


Figure S36. MS (ESI^-^) spectrum of compound **3j**.
